# Constraint-Induced Movement Therapy Versus Bimanual Training to Improve Upper Limb Function in Cerebral Palsy: A Systematic Review and Meta-Analysis of Follow-Ups

**DOI:** 10.3390/children12060804

**Published:** 2025-06-19

**Authors:** Gabriel Martin-Moreno, Marta Moreno-Ligero, Alejandro Salazar, David Lucena-Anton, Jose A. Moral-Munoz

**Affiliations:** 1 Department of Nursing and Physiotherapy, University of Cadiz, 11009 Cádiz, Spain; gabriel.martinmo@alum.uca.es (G.M.-M.); david.lucena@uca.es (D.L.-A.); joseantonio.moral@uca.es (J.A.M.-M.); 2 Department of Statistics and Operational Research, University of Cadiz, 11510 Puerto Real, Spain; alejandro.salazar@uca.es

**Keywords:** cerebral palsy, constraint-induced movement therapy, bimanual therapy, upper limb function

## Abstract

**Background/Objectives**: Constraint-induced movement therapy (CIMT) and bimanual training (BIT) have been commonly used to improve upper limb (ULF) in paediatric populations. This study aimed to compare the efficacy of CIMT and BIT for the recovery of ULF in youth with unilateral cerebral palsy (CP) in the immediate, short, and long term. **Methods**: A systematic review with a meta-analysis of randomised controlled trials (RCTs) from the PubMed/Medline, Scopus, Web of Science, and PEDro databases was conducted. The primary outcomes were the immediate, short-, and long-term effects on ULF, and the secondary outcomes were related to occupational performance and disability. The risk of bias was assessed using the Cochrane RoB 2.0 tool by two researchers independently. Meta-analyses were performed using RevMan 5.3. **Results**: From the 174 records obtained, 10 RTCs comprising 418 participants were included. Favourable results were observed immediately after intervention for CIMT regarding unimanual ULF using the Quality of Upper Extremity Test (QUEST) (SMD = 1.08; 95% CI = (0.66;1.50)) and Jebsen–Taylor Hand Function Test (JTHFT) (SMD = −0.62; 95% CI = (−1.23;0.00)). These results were maintained in the short term for the QUEST for dissociated movements (SMD = 1.19; 95% CI = (0.40;1.99)) and in the long term for the JTHFT (SMD = −0.38; 95% CI = (−1;0.24)). Conversely, favourable results were obtained immediately after the intervention for BIT regarding bimanual ULF using the Assisting Hand Assessment (SMD = −0.42; 95% CI = (−0.78–0.05)). **Conclusions**: CIMT could be more effective for improving unimanual ULF and BIT in youth with unilateral CP. The differences between the interventions decreased in the long term. Nevertheless, these findings should be interpreted with caution due to the variability in the intervention programmes. Further research with standardised protocols is needed.

## 1. Introduction

Cerebral palsy (CP) is a group of movement and postural disorders that affect activity performance [1]. In developed countries, the incidence is estimated to be between 1.4 and 1.8 per 1.000 live births, and it is the most common cause of childhood disability [2]. The development of upper limb function (ULF) is particularly important for independence in activities of daily living (ADL) [3]. The International Classification of Functioning, Disability and Health (ICF) highlights certain abilities as necessary for performing these tasks, such as the ability to move and manipulate objects or fine motor dexterity [4].

In recent years, task-specific training, such as constraint-induced movement therapy (CIMT) and bimanual training (BIT) [5], has gained attention, changing the focus of treatment from structural alterations to interventions based on motor learning. CIMT involves structured intensive practice with the restriction of the less affected upper limb, encouraging the use of the more affected hand to counteract learned non-use [5,6]. BIT also aims to promote the use and coordination of both hands in different activities to improve ULF [7,8].

Previous research has indicated the need for further study on the intensity and efficacy of these interventions in the short and long term. Novak et al. [9] conducted a systematic review and found strong evidence supporting the efficacy of CIMT and BIT for improving upper limb function and the performance of tasks. Dong et al. [10] aimed to compare the efficacy of CIMT and BIT in children with unilateral CP and found that a combination of both interventions had positive effects on improving arm function. Tervahauta et al. [11] also compared the efficacy of CIMT and BIT in children with unilateral CP and concluded that there was limited evidence to support the superiority of one intervention over the other. Finally, Klepper et al. [12] compared unimanual and BIT therapies to improve ULF in children with unilateral CP, finding that BIT provided greater improvements in hand function than unimanual training. Although the previous literature has reported that CIMT and BIT therapies are effective interventions for improving ULF in youth with CP, there have been no studies that specifically compare CIMT with BIT in the immediate, short, and long term.

Therefore, the objective of this systematic review was to compare the efficacy of CIMT and BIT in recovering ULF in terms of motor functioning, occupational performance, and disability at immediate, short-, and long-term follow-ups in youth with CP. We hypothesised that CIMT and BIT therapies could have potential benefits for improving unimanual and bimanual ULF, respectively, in youth with CP.

## 2. Materials and Methods

The Preferred Reporting Items for Systematic Review and Meta-Analysis (PRISMA) 2020 guidelines [13] were used (Supplementary Table S1). Moreover, the review protocol was pre-registered on the International Prospective Register of Systematic Reviews (PROSPERO) database (CRD42022320019).

### 2.1. Search Strategy

The search was performed in the following databases (February–April 2022): PubMed/Medline, Scopus, Web of Science, and the Physiotherapy Evidence Database (PEDro). The search strategy used in each database is described in Supplementary Table S2. The results were limited to randomised controlled trials (RCTs), considered the basis for clinical research on interventions [14]. No other limits were set.

### 2.2. Eligibility Criteria

The PICOS (Population, Intervention, Comparison, Outcomes, Study design) [15] framework was used to establish the following eligibility criteria: (1) youth (<18 years old) with CP (any CP subtype), (2) a comparison between CIMT or modified CIMT (mCIMT) and BIT, including bimanual training activities with intensive practise, (3) the primary outcome was the immediate, short- and long- term effects on ULF (motor function, ability, quality of movement, and strength), with secondary outcomes related to occupational performance and disability, and (4) only RCTs were included.

Studies were excluded from the review if they (1) used a combination of CIMT and BIT therapies or (2) a combination of CIMT and BIT with other medical therapies (e.g., botulinum toxin) and (3) they included other neurological conditions in addition to CP that did not show isolated results.

### 2.3. Study Selection

After retrieving the records from the consulted databases, duplicates were removed using the Rayyan QCRI (Qatar Computing Research Institute) platform [16]. Subsequently, two researchers (G.M.-M. and M.M.-L.) screened the studies according to titles and abstracts. Then, potentially relevant documents were independently reviewed in full-text by G.M.-M. and M.M.-L. according to the pre-established eligibility criteria. We also considered duplicate data from the same cohort or subset cohort of children and selected only one study [6]. A third researcher (J.A.M.-M.) intervened when there was disagreement.

### 2.4. Data Extraction

Data extracted included study characteristics, number of participants, demographic characteristics, study intervention details, primary and secondary outcomes, and outcomes measurements and tools. Moreover, the main findings of intra- and inter-group changes were extracted.

### 2.5. Risk of Bias Assessment

The revised Cochrane risk-of-bias tool (RoB 2.0) [17] was used. This tool is structured into five domains of bias: (1) randomisation process; (2) deviations from the intended interventions; (3) missing outcome data; (4) measurement of the outcome; and (5) selection of the reported result. Each domain was categorised as “low risk”, “high risk”, and “unclear risk”, and an overall assessment was performed. Two researchers (G.M.-M. and M.M.-L.) performed this task, and any disagreements were discussed with a third researcher (J.A.M.-M.).

### 2.6. Statistical Analysis

A meta-analysis was conducted to determine the differences between the interventions. The magnitude of the effect was the standardised mean differences (SMDs) in the changes between groups. Cut-off points of 0.20, 0.50, and 0.80 can be considered to represent a small, moderate, and large effect, respectively [18]. The results are presented with 95% confidence intervals (95%CIs) and a significance level of α = 0.05. Nevertheless, when more than one analysis was joined to lead to a single conclusion, Bonferroni correction was applied to avoid an increase in type I error. In those cases, α was modified to 0.05 divided by the number of comparisons. We considered the presence of heterogeneity if *p* < 0.05 in the chi-square test and the I^2^ statistic was ≥ 50%. This follows the recommendations of the Cochrane Handbook [19]: 0–40% may not be important, 30–60% may represent moderate heterogeneity, 50–90% may indicate substantial heterogeneity, and 75–100% considerable heterogeneity. Subsequently, random-effect (heterogeneity) or fixed-effect (homogeneity) models were used. The risk of publication bias was analysed using Begg’s rank test and funnel plots. Furthermore, sensitivity analyses were performed to evaluate the potential influence of individual study bias on the meta-analyses. The results are presented as forest plots. The timing of the assessment (immediate, short [1–4 months], or long term [over 6 months]), and the measuring instrument in each study determined the different subgroups for the meta-analysis Additionally, we have particularised the analyses for the QUEST and AHA including only studies with more than 90 h of intervention, as this might have and important impact on the results. Studies that used more than one instrument were included in more than one subgroup. All groups compared CIMT with BIT. The Review Manager 5.4 software was used for the analysis. The risk of publication bias was assessed using EPIDAT 3.1.

## 3. Results

### 3.1. Study Selection

From the 174 records obtained, 10 RCTs were included in both the systematic review and meta-analysis (Supplementary Figure S1). The reasons for the exclusion of studies are detailed in Supplementary Table S3.

### 3.2. Data Extraction

A total of 418 participants under 18 years of age with unilateral CP were included, most of whom were diagnosed with spastic hemiplegia (Table 1). Regarding the sources of funding for included RCTs, five [20,21,22,23,24] had some type of funding, two [25,26] reported no funding, and three [27,28,29] did not report this information.

Some differences were observed in the CIMT protocol, such as the type of restraint, which varied among gloves, casts attached to the trunk, or splints. The activities performed by the participants comprised fine and gross motor tasks and games focused on ADL. This was also the objective of the BIT group, but it focused on the coordination of both hands during a task or game (Table 1).

The timing of follow-up assessments varied across studies, although there was a common pattern: baseline intervention assessments and those immediately after the intervention were present in all the articles; in the short-term, we found the assessments were made between 1 and 4 months after the intervention, and in the long-term, most of them were made around the sixth month after the intervention (Table 2). Consequently, the meta-analyses considered the timing of the assessment between comparable interventions, following the criteria of some authors [25,30,31,32] who distinguished between three terms: immediate term (right after the intervention), long term (6 months after the intervention onwards), and short term (between these two).

### 3.3. Risk of Bias Assessment

**Figure 1:**
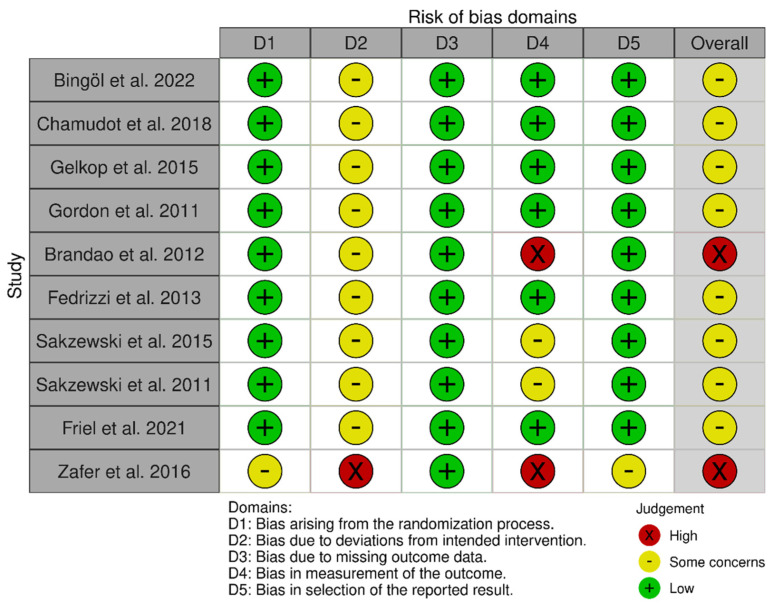
Risk of bias for each included study [20,21,22,23,24,25,26,27,28,29].

**Figure 2:**
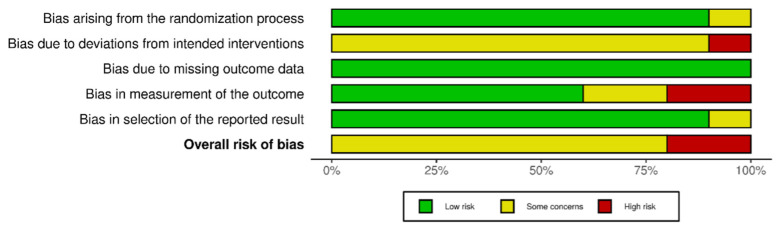
Summary of the assessment for each bias.

Regarding the risk of bias, most studies were classified as having some concerns, except for two [21,26], which were classified as high-risk. For a detailed analysis of the evidence synthesis, see Figure 1 and Figure 2.

### 3.4. Synthesis of Results

Based on the primary and secondary outcomes, the results were divided into three categories: (i) immediate, (ii) short-term effects, and (iii) long-term effects. In addition, the results are presented according to the outcome analysed. The different groups and subgroups obtained by meta-analysis, including the studies, measuring instruments, and outcome measures, are shown in Table 3.

In addition, the results of the QUEST and AHA were particularised, including only studies with more than 90 h of intervention.

#### 3.4.1. Immediate, Short-Term, and Long-Term Effects

##### Immediate Term

- Unimanual upper limb function.

Five studies used the QUEST to assess unimanual ULF. The studies by Gelkop et al. [29], Bingöl et al. [27], and Zafer et al. [26] obtained the best results for the QUEST dissociated movement domain, grasp domain, and QUEST total score, respectively. Regarding the results on unimanual ULF immediately after intervention through the QUEST, CIMT showed a greater improvement in the quality of movement for the dissociated movement domain (SMD = 0.92; 95%CI = (0.51;1.33)), grasp domain (SMD = 1.20; 95%CI = (0.31;2.09)) and the QUEST total score (SMD = 1.08; 95%CI = (0.66;1.50)).

**Figure 3:**
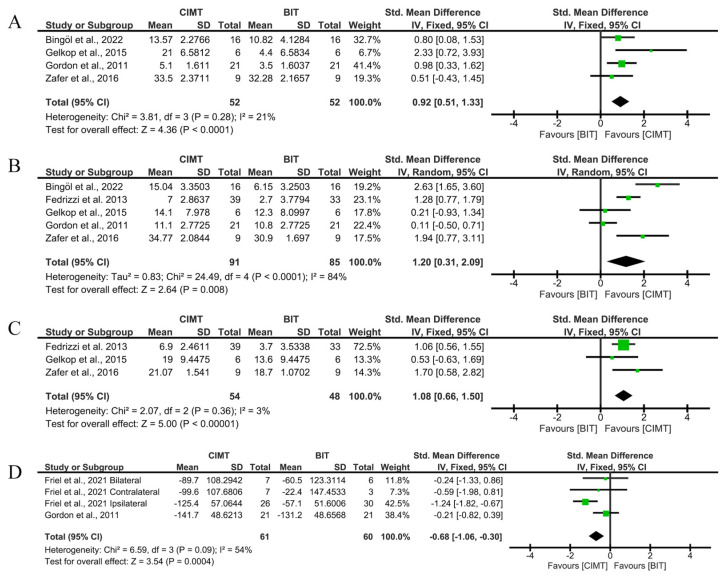
Immediate-term results for unimanual UFL measured using QUEST and JTHFT. (**A**) Std. mean difference (95% CI) of the effect of CIMT vs. BIT in the QUEST dissociated movement domain [20,26,27,29]; (**B**) std. mean difference (95% CI) of the effect of CIMT vs. BIT in the QUEST grasp domain [20,25,26,27,29]; (**C**) std. mean difference (95% CI) of the effect of CIMT vs. BIT in total QUEST [25,26,29]; (**D**) std. mean difference (95% CI) of the effect of CIMT vs. BIT on the JTHFT [20,24]. BIT: bimanual training; CI: confidence interval; CIMT: constraint-induced movement therapy; IV: inverse variance; SD: standard deviation. Bonferroni corrected significance level: α = 0.05/4 = 0.0125. The green squares represent the effect estimate for each study. Size is proportional to the study weight in the group. Black diamonds represent the pooled effect. The widths represent the 95% CI of the combined result.

Two studies used the JTHFT to assess unimanual ULF, with Friel et al. [24] achieving the best results. Regarding the immediate results on unimanual ULF through the JTHFT, the same results were found in favour of CIMT when compared to BIT (SMD = −0.62; 95%CI = (−1.23;0.00)). The interpretation of the SMD suggests large effects on QUEST, but moderate effects on JTHFT. The statistical significance of the results remained after Bonferroni correction was applied for multiple comparisons in all cases (Figure 3).

No risk of publication bias was observed in any of the analyses (Begg’s test *p* = 0.7341 in Figure 3A, *p* = 0.8065 in Figure 3B; *p* > 0.999 in Figure 3C and *p* = 0.7341 in Figure 3D). Funnel plots are available in Supplementary Figures S2.1–S2.4. Supplementary Figures S3.1–S3.3 show the sensitivity analyses, excluding a paper with a high risk of bias [26]. No significant changes were observed in the global results.

- Bimanual upper limb function.

**Figure 4:**
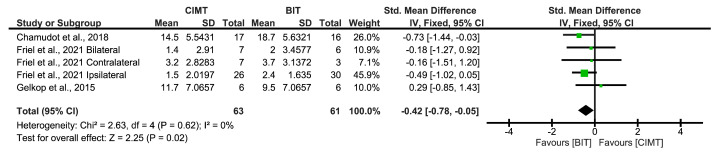
Immediate-term results for bimanual UFL measured using AHA [24,28,29]. BIT: bimanual training; CI: confidence interval; CIMT: constraint-induced movement therapy; IV: inverse variance; SD: standard deviation. The green squares represent the effect estimate for each study. Size is proportional to the study weight in the group. Black diamonds represent the pooled effect. The widths represent the 95% CI of the combined result.

Three studies used the AHA scale to assess the bimanual ULF. The best results were reported by Chamudot et al. [28]; contrary to what was observed with the unimanual ULF, BIT showed better results than CIMT for the bimanual ULF through the AHA (SMD = −0.42; 95%CI = (−0.78;−0.05)), although this effect may be considered moderate (Figure 4).

The results of Begg’s rank test showed no evidence of a risk of publication bias (*p* = 0.4624). The funnel plot is shown in Supplementary Figure S2.5.

- Occupational performance and disability.

Regarding occupational performance and disability, the meta-analyses were conducted only to assess immediate effects after the intervention. Four studies used the COPM instrument to assess occupational performance, with Brandao et al. [21] and Sakzewski et al. [22] achieving the best results. The overall results of the COPM were not conclusive, although they showed a tendency in favour of BIT: performance (SMD = −1.23; 95%CI = (−2.60;0.14)) and satisfaction (SMD = −0.93; 95%CI = (−1.98;0.12)).

**Figure 5:**
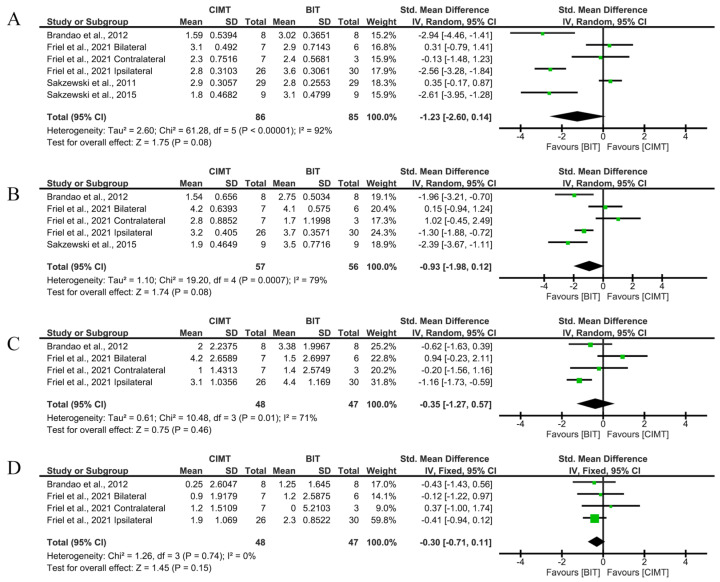
Immediate-term results for occupational performance and disability measured by COPM and PEDI. (**A**) Std. mean difference (95% CI) of the effect of CIMT vs. BIT in the COPM performance domain [21,22,23,24]; (**B**) std. mean difference (95% CI) of the effect of CIMT vs. BIT in the COPM satisfaction domain [21,22,24]; (**C**) std. mean difference (95% CI) of the effect of CIMT vs. BIT in the PEDI functional skills domain [21,24]; (**D**) std. mean difference (95% CI) of the effect of CIMT vs. BIT in the PEDI independence domain [21,24]. BIT: bimanual training; CI: confidence interval; CIMT: constraint-induced movement therapy; IV: inverse variance; SD: standard deviation. Bonferroni corrected significance level: α = 0.05/4 = 0.0125. The green squares represent the effect estimate for each study. Size is proportional to the study weight in the group. Black diamonds represent the pooled effect. The widths represent the 95% CI of the combined result.

Two studies used the PEDI scale to assess disability, with Friel et al. [24] achieving the best results. No significant results were found for the comparison between CIMT and BIT for the PEDI (SMD = −0.3; 95%CI = (−0.71;0.11)). Given the absence of statistical significance (even before Bonferroni corrections), we do not recommend the interpretation of the intensity of the effects depending on the SMD (Figure 5).

None of the analyses showed a risk of publication bias (*p* = 0.4524 in Figure 5A, *p* = 0.8065 in Figure 5B, *p* = 0.3082 in Figure 5C and *p* = 0.0894 in Figure 5D). Funnel plots are available in Supplementary Figures S2.6–S2.9. Sensitivity analyses, excluding the paper with a higher risk of bias [21], are shown in Supplementary Figures S3.4–S3.7. The global results show no relevant changes.

##### Short Term

- Unimanual upper limb function.

Four studies used the QUEST to assess the short-term effects of unimanual ULF. The studies by Gordon et al. [20], Bingöl et al. [27], and Fedrizzi et al. [25] obtained the best results in the QUEST dissociated movement domain, grasp domain, and QUEST total score, respectively. CIMT showed a greater improvement than BIT for dissociated movements (SMD = 1.2; 95%CI = (0.73;1.68)), but not in grasp (SMD = 0.95; 95%CI = (−0.01;1.91)) after correcting the significance level. The former may be considered a large effect, according to the SMD value. The total score analysis did not show a better result than the BIT (SMD = −0.16; 95%CI = (−0.59;0.27)).

**Figure 6:**
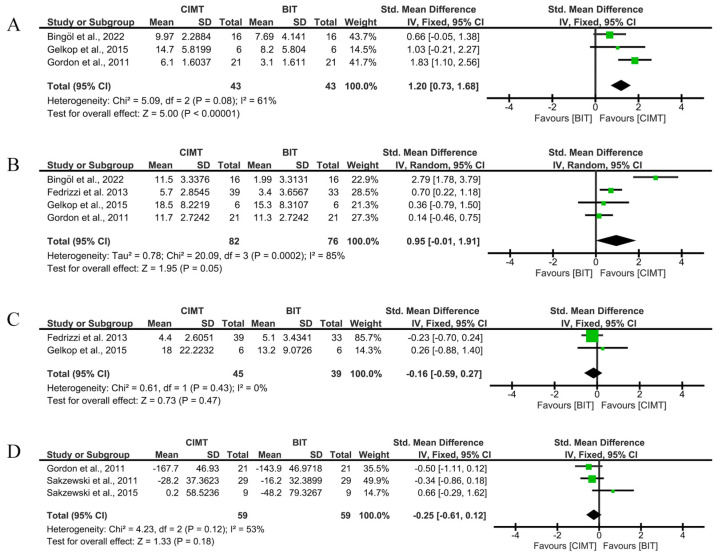
Short-term results for unimanual UFL measured by QUEST and JTHFT. (**A**) Std. mean difference (95% CI) of the effect of CIMT vs. BIT in the QUEST dissociated movement domain [20,27,29]; (**B**) std. mean difference (95% CI) of the effect of CIMT vs. BIT in the QUEST grasp domain [20,25,27,29]; (**C**) std. mean difference (95% CI) of the effect of CIMT vs. BIT in the total QUEST [25,29]; (**D**) std. mean difference (95% CI) of the effect of CIMT vs. BIT on the JTHFT [20,22,23]. BIT: bimanual training; CI: confidence interval; CIMT: constraint-induced movement therapy; IV: inverse variance; SD: standard deviation. Bonferroni corrected significance level: α = 0.05/4 = 0.0125. The green squares represent the effect estimate for each study. Size is proportional to the study weight in the group. Black diamonds represent the pooled effect. The widths represent the 95% CI of the combined result.

Three studies used the JTHFT to assess unimanual ULF, with Sakzewski et al. [23] achieving the best results. The overall result showed no significant differences between therapies (SMD = −0.17; 95%CI = (−0.73;0.39) (Figure 6).

According to the results, no risk of publication bias was observed (*p* > 0.999 in Figure 6A, *p* = 0.7341 in Figure 6B, *p* > 0.999 in Figure 6C, and *p* > 0.999 in Figure 6D). Funnel plots are available in Supplementary Figures S2.10–S2.12.

- Bimanual upper limb function.

**Figure 7:**
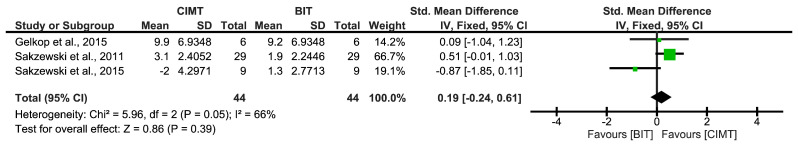
Short-term results for bimanual ULF measured by the AHA [22,23,29]. BIT: bimanual training; CI: confidence interval; CIMT: constraint-induced movement therapy; IV: inverse variance; SD: standard deviation. The green squares represent the effect estimate for each study. Size is proportional to the study weight in the group. Black diamonds represent the pooled effect. The widths represent the 95% CI of the combined result.

Three studies used the AHA scale to assess the short-term effects on bimanual ULF, with Sakzewski et al. [22] achieving the best results. The overall result showed no significant differences between therapies (SMD = −0.02; 95%CI = (−0.88;0.83)) (Figure 7).

No risk of publication bias was observed (*p* > 0.999). The funnel plot is shown in Supplementary Figure S2.13.

##### Long Term

- Unimanual upper limb function.

Two studies assessed the long-term effects on unimanual ULF through the QUEST grasp domain, with Gordon et al. [20] obtaining the best results. The overall result of the meta-analysis showed no conclusive results between interventions (SMD = 0.15; 95%CI = (−0.55;0.85)).

**Figure 8:**
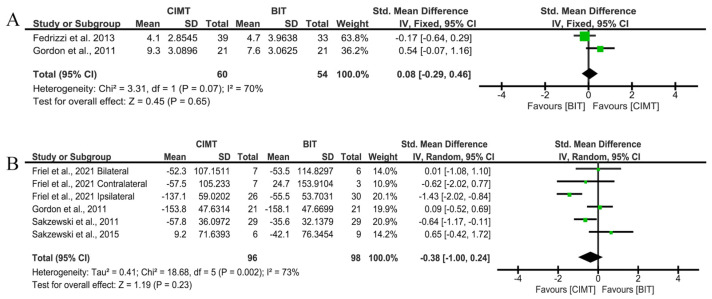
Long-term results for unimanual ULF measured by QUEST and JTHFT. (**A**) Std. mean difference (95% CI) of the effect of CIMT vs. BIT in the QUEST grasp domain [20,25]; (**B**) std. mean difference (95% CI) of the effect of CIMT vs. BIT on the JTHFT [20,22,23,24]. BIT: bimanual training; CI: confidence interval; CIMT: constraint-induced movement therapy; IV: inverse variance; SD: standard deviation. Bonferroni corrected significance level: α = 0.05/2 = 0.025. The green squares represent the effect estimate for each study. Size is proportional to the study weight in the group. Black diamonds represent the pooled effect. The widths represent the 95% CI of the combined result.

Four studies used the JTHFT to assess unimanual ULF, with Friel et al. [24] achieving the best results. No significant results were found for CIMT in the JTHFT (SMD = −0.38; 95%CI = (−1;0.24)) (Figure 8).

In these cases, no risk of publication bias was observed (*p* > 0.999 in Figure 8A and *p* = 0.7071 in Figure 8B). The funnel plot is shown in Supplementary Figure S2.14.

- Bimanual upper limb function.

**Figure 9:**
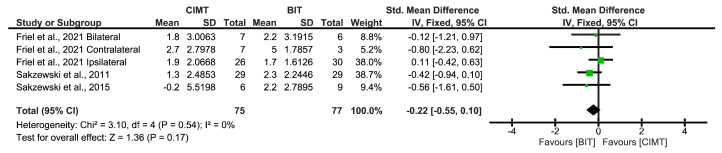
Long-term results for bimanual ULF measured by the AHA [22,23,24]. BIT: bimanual training; CI: confidence interval; CIMT: constraint-induced movement therapy; IV: inverse variance; SD: standard deviation. The green squares represent the effect estimate for each study. Size is proportional to the study weight in the group. Black diamonds represent the pooled effect. The widths represent the 95% CI of the combined result.

Concerning the bimanual ULF, three studies used the AHA scale. Friel et al. [24] obtained the best results. No conclusive results between interventions were found (SMD = 0.08; 95%CI = (−0.29;0.46)) (Figure 9).

Finally, no risk of publication bias was identified (*p* = 0.8065). The funnel plot is shown in Supplementary Figure S2.15.

#### 3.4.2. Total Intervention Hours (>90)

**Figure 10:**
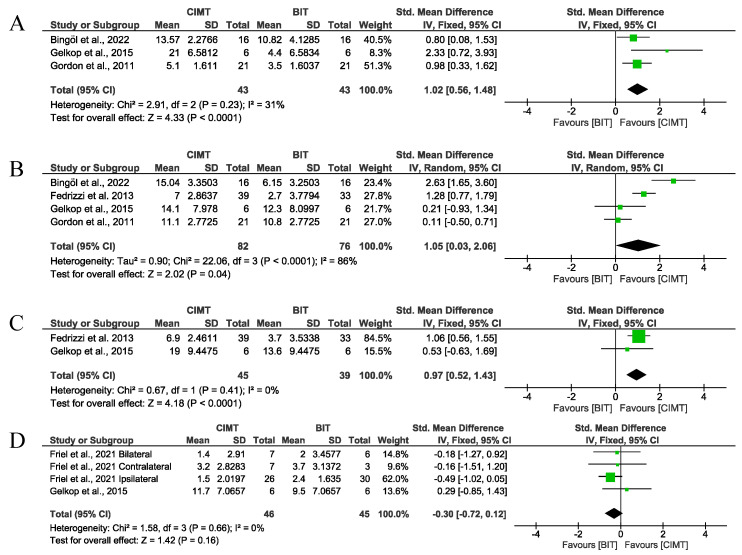
Results for unimanual ULF measured by QUEST and JTHFT. (**A**) Std. mean difference (95% CI) of the effect of CIMT vs. BIT in the QUEST dissociated movement domain [20,27,29]; (**B**) std. mean difference (95% CI) of the effect of CIMT vs. BIT in the QUEST grasp domain [20,25,27,29]; (**C**) std. mean difference (95% CI) of the effect of CIMT vs. BIT in the total QUEST [25,29]; (**D**) std. mean difference (95% CI) of the effect of CIMT vs. BIT on the JTHFT [24,29]. BIT: bimanual training; CI: confidence interval; CIMT: constraint-induced movement therapy; IV: inverse variance; SD: standard deviation. Bonferroni corrected significance level: α = 0.05/4 = 0.0125. The green squares represent the effect estimate for each study. Size is proportional to the study weight in the group. Black diamonds represent the pooled effect. The widths represent the 95% CI of the combined result.

Finally, when particularising the results for unimanual ULF only in cases where the intervention lasted at least 90 h, four studies measured it using the QUEST and two studies using the JTHFT. CIMT again showed a greater improvement than BIT for dissociated movements (SMD = 1.02; 95%CI = (0.56;1.48)), being considered a large effect given the SMD value. However, the results were not statistically significant for grasping (*p* > α = 0.0125 after Bonferroni correction). Nonetheless, this time, the total score showed a better result (large effect) than the BIT (SMD = 0.97; 95%CI = (0.52;1.43)), considering only interventions over 90 h, even after applying Bonferroni correction. For the JTHFT, no significant differences were observed between therapies (Figure 10).

According to the results, no risk of publication bias was observed (*p* > 0.999 in Figure 10A, *p* = 0.7341 in Figure 10B, *p* > 0.999 in Figure 10C, and *p* = 0.7341 in Figure 10D). Funnel plots are available in Supplementary Figures S2.16–2.17.

## 4. Discussion

This study aimed to determine the immediate, short-, and long-term effects of CIMT compared with BIT among youths with CP on ULF, occupational performance, and disability. To our knowledge, this study is the first to conduct a meta-analysis of RCTs comparing CIMT with BIT. CIMT showed superior efficacy in terms of unimanual ULF measured through the QUEST and JTHFT immediately after intervention, maintaining these results in the short-term for the QUEST dissociated movements, but not for the rest of the dimensions. Conversely, BIT showed significant results for bimanual ULF measured through the AHA immediately after the intervention. No conclusive results were found for occupational performance and disability. Although some pooled effect sizes were statistically significant, these results should be interpreted with caution. In several cases, the SMD showed moderate or even large effects statistically, but their clinical relevance remains uncertain.

Furthermore, before discussing the results obtained, it is important to highlight the variability in the intervention protocols. The total dosage ranged from 24 to 125 h, while delivery formats varied from short daily home sessions (e.g., 1 h/day, 7 days/week) to intensive day camps (e.g., 6 h/day over consecutive days). Furthermore, specific therapeutic activities and strategies vary widely, encompassing fine and gross motor tasks, games, manipulation, and balance training. In that sense, a review exploring CIMT programmes for CP reported diverse frequency, intensity, and duration parameters, underscoring challenges in identifying an optimal dosage for motor function gains [33]. This clinical and methodological heterogeneity introduces potential confounders and selection bias, thereby reducing the validity and generalisability of the study results. This also likely contributed to the statistical heterogeneity observed in the meta-analysis. This diversity underscores the lack of standardised protocols in rehabilitation, limiting the generalisability of the pooled results and raising questions about the optimal dosage, modality, and setting for specific patient profiles.

Concerning our results obtained on unimanual ULF, CIMT was more effective than BIT for improving unimanual ULF in the immediate term. These results agreed with previous systematic reviews [10,34], although they did not analyse the follow-up effects and did not compare CIMT with BIT. Furthermore, other systematic reviews [11,12] concluded that there was no superiority of CIMT over BIT in both the short and long term, but a statistical comparison was not performed. Regarding our results, the superiority of CIMT in the immediate term could be explained by well-structured protocols. BIT did not have similar structuring, except for the Hand–Arm Bimanual Intensive Training (HABIT) protocol, which was used in 4/10 RCTs included [20,21,24,29]. Beyond the role of structured protocols, neurophysiological and motor-learning mechanisms likely contribute to CIMT superiority in unimanual ULF. In paediatric CP, structural neuroplastic changes are observed following intensive CIMT. For instance, Sterling et al. [35] reported significant grey matter increases in the sensorimotor cortex contralateral to the affected limb, which were correlated with function. Similarly, studies using functional near-infrared spectroscopy and fNIRS have shown altered activation patterns and increased functional connectivity in sensorimotor regions post CIMT, persisting up to six months after intervention [36]. These findings align with the principles of use-dependent plasticity and motor learning, such as intensity, repetition, and specificity, and provide biological plausibility underpinning the observed improvements in unimanual ULF with CIMT.

Regarding bimanual ULF, previous systematic reviews [10,12] have stated that BIT tends to have better results than CIMT. Our results agree with this in the immediate term. Children with unilateral CP present with more than just limited mobility; their ability to coordinate both hands spatially and temporally is also impaired, in addition to difficulties with motor planning [8]. In this sense, the integral components of BIT are the use of the impaired upper limb to stabilise, grasp, or manipulate an object during bimanual activities, incorporating the child’s objectives and extensive family engagement, and the prioritisation of active problem-solving during practise [12]. Therefore, BIT is better for achieving bimanual coordination and goals and enhancing the transfer of unpractised tasks [10].

Regarding occupational performance and disability, we found no statistical differences between BIT and CIMT. Nevertheless, BIT is a specific training approach focused on using both hands, similar to the ADL. Then, we hypothesised that improvements in bimanual ULF obtained by the BIT group could influence occupational performance and disability, but this is not supported by our findings. Although the results did not reach statistical significance, outcomes such as COPM performance and satisfaction showed moderate-to-large effect sizes in favour of BIT. This suggests potential clinical relevance that may not have been fully captured due to insufficient statistical power or sample size and should be explored in future studies. It was previously stated that the bimanual approach can have a greater influence on daily routines, meaningful goals, and transfer to unpractised tasks [8,10]. Furthermore, the comparison was only possible in the immediate term, so it would be interesting to explore the levels of improvement in longer follow-up in future studies.

Regarding long-term effects, the differences between interventions were attenuated over time, and no statistically significant results were observed. Nonetheless, some trends favouring CIMT for unimanual function, as measured by the JTHFT, and BIT for bimanual coordination, assessed by the AHA, were identified. These findings suggest that the relative benefits of each intervention may evolve over time and highlight the need for longer follow-up periods in future trials.

The duration and frequency of the intervention may have influenced the results obtained. Regarding duration, 6/10 studies performed over 90 h of intervention and 4/10 performed less than 90 h [22,23,26,28]. In this line, regarding the immediate-term effects on unimanual ULF, most interventions achieved superiority for CIMT over 90 h, which could be explained by the initial increase in attention given to the affected limb and the lack of compensation because of restraint [10,29]. It is also notable that 5/10 studies performed a less intensive but more extended (8–10 weeks) intervention, with lower frequency (3 h, 3 times/week or 1–2 h/day), while 5/10 studies chose a more intensive intervention with a less extended protocol (1–2 weeks) and higher frequency (6 h/day). High frequencies and durations are essential as they have been correlated with improvement in youth with CP, with the total programme duration being more relevant when the intervention is a structured practise [37]. In this way, in our study, when only studies that provided more than 90 h of intervention were considered, CIMT continued to show greater improvements in ULF as measured by the QUEST, particularly in dissociated movements and total scores. These findings suggest a possible dose–response relationship, where a higher intensity or longer duration may enhance the efficacy of CIMT. However, the superiority of CIMT was not evident in the JTHFT when considering the same dosage threshold, which may indicate task-specific benefits rather than global improvements. These results should be interpreted with caution because the number of included studies in this subgroup analysis was limited, and the variability in protocols remains high.

Moreover, subgroup analyses based on follow-up timing or intervention dosage are often limited by small sample sizes, reduced statistical power, and an increased risk of type II error. These analyses should be interpreted with caution, as the findings may not reflect robust subgroup effects, but rather random variation.

Finally, concerning the risk of bias, most studies were classified as having some concerns regarding their methodological rigour. This could be attributed to factors such as participants having knowledge of the intervention received, a lack of blinding in some outcome assessments, and the absence of intention-to-treat analyses. Furthermore, methodological designs and a lack of consensus regarding protocols for the same intervention are possible causes of heterogeneity. These studies are usually conducted in clinical or home-based settings without controlling for environmental influences and involve institutionalised patients. Therefore, selection bias may be introduced by the need to conduct multicentre studies without convenience samples [38]. Thus, all necessary measures were taken to ensure the correct synthesis of results. On the one hand, no publication bias was found in any of the meta-analyses obtained, reinforcing the results obtained. On the other hand, the potential impact of the risk of bias in individual studies on the results of the meta-analyses was assessed using sensitivity analyses. With the removal of studies with a high risk of bias, the results of the meta-analyses were consistent. Therefore, we did not find any factors that affected the interpretation of our results.

From a clinical perspective, these findings suggest that both CIMT and BIT can be beneficial; however, the selection of one approach over another should be tailored to the patient’s specific functional goals, preferences, and treatment context. The lack of consistency across studies underscores the need for individualised rehabilitation planning. Additionally, the absence of standardised protocols limits the implementation of generalised recommendations in clinical practice. Future clinical trials should aim to determine which patient profiles benefit most from each type of intervention and under what conditions.

### Limitations and Strengths

Some limitations should be acknowledged. First, variability in intervention programmes was high, which, combined with very small subgroup sample sizes, limited the certainty of our pooled estimates and the reliability of the interaction tests. Second, the current volume of studies may limit the generalisability of the findings because the heterogeneity of follow-up assessments influences consistency and comparability. The methodological quality of some RCTs was also compromised, with concerns such as deviations from intended interventions, a lack of blinding, and potential biases because of the knowledge of the interventions received.

Nevertheless, this study has several notable strengths. First, including RCTs only strengthened the study’s evidence base and minimised the influence of confounding variables. Second, the findings have significant implications for clinical practice, offering evidence-based insights to guide clinician decision-making. Finally, this study synthesised multiple studies, providing an overview of the current evidence on CIMT compared with BIT for ULF in youth with unilateral CP.

## 5. Conclusions

CIMT was found to be more effective than BIT in immediately enhancing unimanual ULF, which was also maintained in the short-term for the QUEST dissociated movements, but not for the rest of the dimensions. BIT yielded significant results for bimanual ULF immediately after intervention. Although not statistically significant, outcomes such as COPM showed moderate-to-large effect sizes in favour of BIT, suggesting potential clinical relevance that warrants further investigation. In the long-term, the differences between interventions diminished, with no statistically significant results for either CIMT or BIT, although some trends in favour of CIMT for unimanual function (JTHFT) and BIT for bimanual use (AHA) were observed. Future research should further explore the optimal duration and frequency of interventions as these factors appear to influence the results.

Regarding our results, clinicians and therapists should consider the specific outcomes that they aim to improve when selecting CIMT or BIT. Given the high variability in intervention programmes across the included RCTs, these pooled estimates should be interpreted with caution; adequately powered, harmonised-protocol trials are still required to confirm the differences between CIMT and BIT for this population. Therefore, in view of this heterogeneity, considering the individual’s needs and goals may be the most effective strategy in neurological rehabilitation for young people with CP.

## Figures and Tables

**Table 1:** Participants and intervention protocols from the studies included in the review.

Study	Participants			Intervention Protocol (Duration and Frequency)	
	Total N Gender Proportion	Mean Age	PC Type	Unimanual	Bimanual
Bingöl et al. [27], 2022 Turkey	N = 32 (16/16); Girls/boys: 15/17	Age (years, months): 10.43 ± 2.9	Spastic hemiplegia	mCIMT: restriction with glove, gross and fine motor tasks, and manipulation. 2.5 h/day; 3 days/week; 10 weeks + home: 1 h/day; 5 days/week; 10 weeks; 125 h.	BIT: gross motor tasks regarding bimanual coordination. 2.5 h/day; 3 days/week; 10 weeks + home: 1 h/day; 5 days/week; 10 weeks; 125 h.
Chamudot et al. [28], 2018 Israel	N = 33 (17/16); Girls/boys: 14/19	Age (months): 11.1 ± 2.2	Spastic hemiplegia	mCIMT: at home, restriction with glove, functional activities/tasks. 1 h/day; 7 days/week; 8 weeks; 46.7 h ± 9.9	BIT: at home, symmetric and asymmetric bimanual tasks. 1 h/day; 7 days/week; 8 weeks; 46.7 h ± 9.9
Gelkop et al. [29], 2015 Israel	N = 12 (6/6); Girls/boys: 10/2	Unimanual group: 4.25 ± 1.58 Bimanual group: 4.33 ± 1.86	Spastic hemiplegia	mCIMT: restriction with glove, gross and fine motor tasks, fragmented. 2 h/day (1 h individual/1 h group; 6 days/week; 8 weeks; 96 h	HABIT: gross and fine motor bimanual tasks. 2 h/day (1 h individual/1 h group; 6 days/week; 8 weeks; 96 h
Gordon et al. [20], 2011 USA	N = 42, (21/21); Girls/boys: 22/20	Age (years, months) Unimanual group: 6.3 ± 2.2 Bimanual group: 6.4 ± 1.11	Congenital hemiplegia	mCIMT: day camp, restriction with sling, gross and fine motor tasks. 6 h/day; 15 consecutive days; 90 h	HABIT: gross and fine symmetric and asymmetric motor tasks. 6 h/day; 15 consecutive days; 90 h
Brandao et al. [21], 2012 Brazil	N = 16 (8/8); Girls/boys: 6/10	Age (years, months) Unimanual group: 6.11 ± 2.12 Bimanual group: 6.55 ± 2.13	Hemiplegia	mCIMT: day camp, restriction with sling, gross and fine motor tasks. 6 h/day; 15 consecutive days; 90 h	HABIT: gross and fine symmetric and asymmetric motor tasks. 6 h/day; 15 consecutive days; 90 h
Fedrizzi et al. [25], 2013 Italy	N = 105 (39/33/33)	Age (years, months): 4 ± 8	Hemiplegia	CIMT: restriction with glove and plastic splint, manipulation, grasp, balance, and functional tasks. 3 h/day; 3 days/week; 10 weeks; 90 h	BIT: bimanual manipulation, grasp, balance, and functional tasks. 3 h/day; 3 days/week; 10 weeks; 90 h
Sakzewski et al. [22], 2015 Australia	N = 17 (8/9)	Age (years, months) Unimanual group: 8.5 ± 1.5 Bimanual group: 8.9 ± 1.5	Spastic hemiplegia	mCIMT: group, day camp, restriction with glove and splint, gross and fine motor tasks, functional activities, and games. 6 h/day; 5 consecutive days; 30 h	BIT: group, day camp, bimanual coordination repetitive tasks, and gross and fine motor tasks. 6 h/day; 5 consecutive days; 30 h
Sakzewski et al. [23], 2011 Australia	N = 64 (26/26); Girls/boys: 31/33	Age (years, months): 10.2 ± 2.7	Spastic hemiplegia	CIMT: group, day camp, restriction with glove and splint, gross and fine motor tasks, functional activities, and games. 6 h/day; 10 consecutive days; 60 h	BIT: group, day camp, bimanual coordination repetitive tasks, gross and fine motor tasks. 6 h/day; 10 consecutive days; 60 h
Friel et al. [24], 2021 USA	N = 79 (40/39); Girls/boys: 31/48	Age (years, months) Unimanual group: 9.4 ± 2.10 Bimanual group: 9.7 ± 3.5	Spastic hemiplegia	CIMT: restriction with sling, gross and fine motor functional tasks. 6 h/day; 15 consecutive days; 90 h	HABIT: gross and fine motor bimanual tasks. 6 h/day; 15 consecutive days; 90 h
Zafer et al. [26], 2016 Pakistan	N = 18 (9/9); Girls/boys: 3/15	Age (years, months): 8.75 ± 3.06	Spastic hemiplegia	CIMT: at home, restriction with glove and sling, reach, grasp, and weight bearing tasks. 2 h/day; 6 days/week; 2 weeks; 24 h	BIT: at home, bimanual reach, grasp, and weight bearing tasks. 2 h/day; 6 days/week; 2 weeks; 24 h

Abbreviations: BIT = bimanual training; CIMT = constraint-induced movement therapy; h = hours; HABIT = Hand–Arm Bimanual Intensive Training; mCIMT = modified constraint-induced movement therapy; N = sample size.

**Table 2:** Outcomes, assessment, follow-up, and results from the studies included in the review.

Study	Outcomes	Assessment Tools	Follow-Up	Results (<0.05)	
				Between Groups	Intragroups
Bingöl et al. [27], 2022 Turkey	Primary: Unimanual ability Grasp strength Secondary: Occupational performance and participation	QUEST Dynamometry ABILHAND-Kids CHEQ CASP PMAL	Before IT; after IT; 16 weeks.	Grasp strength: mCIMT (*p* < 0.001) Unimanual ability: mCIMT (*p* < 0.001) and in the follow-up (*p* < 0.001) Bimanual hand use: BT (*p* < 0.001). Affected limb use: mCIMT (*p* < 0.001). Participation: mCIMT (*p* < 0.001).	-
Chamudot et al. [28] 2018 Israel	Primary: Manual function Gross motor function Secondary: Motivation	mini AHA FI DMQ	Before and after IT	No significant differences between groups.	Both in mini AHA and FI (*p* < 0.01).
Gelkop et al. [29], 2015 Israel	Primary: Bimanual function Upper limb function Secondary: Not specified	AHA QUEST	2 months before IT; before IT; after IT; 2-month follow-up.	Dissociated movements in CIMT (*p* < 0.05) before and after IT.	Both in AHA (*p* < 0.05) and QUEST (*p* < 0.05).
Gordon et al. [20], 2011 USA	Primary: Upper limb function Secondary: Dissociated movements Grasp Goal attainment	AHA JTHFT QUEST GAS	Before IT; 2 days after IT; 1-month follow-up; 6-month follow-up.	HABIT in goal attainment and transfer to non-established goals (*p* < 0.05).	Both in QUEST (*p* < 0.001) and JTHFT (*p* < 0.001).
Brandao et al. [21], 2012 Brazil	Primary: Functional independence Caregiver assistance Occupational performance Secondary: Not specified	PEDI COPM	Before and after IT.	No significant differences between groups.	Both in PEDI functional independence (*p* = 0.0001) and caregiver assistance (*p* = 0.01). Both in COPM performance (*p* < 0.000) and satisfaction (*p* = 0.0001). Both in practised goals (*p* = 0.003) and unpractised (*p* = 0.0001)
Fedrizzi et al. [25], 2013 Italy	Primary: Upper limb function Secondary: Not specified	QUEST Besta Scale	Before IT; after IT; 3-month follow-up; 6-month follow-up.	BIT in Besta Scale (*p* = 0.0139). -CIMT affected hand (*p* = 0.0019); BIT in the follow-ups (*p* = 0.0270). BIT non-affected hand (*p* = 0.0280); CIMT (*p* = 0.0154) at 3-month follow-up.	Both in QUEST and Besta Scale (*p* < 0.001)
Sakzewski et al. [22], 2015 Australia	Primary: Quality of movement of the upper limb Bimanual function Secondary: Occupational performance Speed and dexterity of the upper limb	MUUL AHA CPQOL JTHFT COPM	Before IT; 3 weeks after IT; 26-week follow-up.	No significant differences between groups.	Both (*p* < 0.005) in COPM
Sakzewski et al. [23], 2011 Australia	Primary: Quality of movement of the upper limb Bimanual function Secondary: Occupational performance Speed and dexterity of the upper limb	MUUL AHA CPQOL JTHFT COPM	Before IT; 3 weeks after IT; 26-week follow-up; 1-year follow-up.	CIMT in unimanual capacity (*p* = 0.005).	CIMT in quality and efficacy of movement at 26-week and 1-year follow-ups (*p* < 0.001). BIT in efficacy between 26-week and 1-year follow-ups (*p* = 0.001). Both in COPM (*p* < 0.001) 1-year follow-up.
Friel et al. [24], 2021 USA	Primary: Manual function and laterality of the corticospinal tract Secondary: Occupational performance Goal attainment	AHA JTHFT BBT COPM ABILHAND-kids PEDI	Before IT; after IT; 6 month follow-up.	Both in AHA, JTHFT and BBT (*p* < 0.001) maintained at 6 months.	Both in hand use in ADLs, COPM and PEDI (*p* < 0.001).
Zafer et al. [26], 2016 Pakistan	Primary: Upper limb function Secondary: Not specified	QUEST	Before and after IT.	CIMT in dissociated movements, grasp and QUEST total (*p* < 0.05).	Both in dissociated movements and grasp (*p* < 0.05).

Abbreviations: AHA = Assisting Hand Assessment; ADL = activities of daily life; BBT = Box and Block Test; BIT = bimanual training; CASP = Child and Adolescent Scale of Participation; CHEQ = Children’s Hand-Use Experience Questionnaire; CIMT = constraint-induced movement therapy; COPM = Canadian Occupational Performance Measure; CPQOL = Cerebral Palsy Quality of Life Questionnaire; DMQ = Dimensions of Mastery Questionnaire; FI = Functional Inventory; GAS = Goal Attainment Scale; h = hours; HABIT = Hand–Arm Bimanual Intensive Training; IT = intervention; JTHFT = Jebsen–Taylor Hand Function Test; mCIMT = modified constraint-induced movement therapy; MUUL = Melbourne Assessment of Unilateral Upper Limb Function; PEDI = Paediatric Evaluation of Disability Inventory; PMAL = Paediatric Motor Activity Log; QUEST = Quality of Upper Extremity Test.

**Table 3:** Study groups included in the meta-analysis according to time effects.

Time Effects	Outcome Measures	Measuring Instruments	Studies
Immediate	Unimanual ULF	QUEST dissociate domain	Bingöl et al., 2022 [27]; Gelkop et al., 2015 [29]; Gordon et al., 2011 [20]; Zafer et al., 2016 [26]
		QUEST grasp domain	Bingöl et al., 2022 [27]; Fedrizzi et al., 2013 [25]; Gelkop et al., 2015 [29]; Gordon et al., 2011 [20]; Zafer et al., 2016 [26]
		QUEST total score	Fedrizzi et al., 2013 [25]; Gelkop et al., 2015 [29]; Zafer et al., 2016 [26]
		JTHFT	Friel et al., 2021 [24] Bilateral; Friel et al., 2021 [24] Contralateral; Friel et al., 2021 [24] Ipsilateral; Gordon et al., 2011 [20]
	Bimanual ULF	AHA	Chamudot et al., 2018 [28]; Friel et al., 2021 [24] Bilateral; Friel et al., 2021 [24] Contralateral; Friel et al., 2021 [24] Ipsilateral; Gelkop et al., 2015 [29]
	Occupational performance and disability	COPM Performance	Brandao et al., 2012 [21]; Friel et al., 2021 [24] Bilateral; Friel et al., 2021 [24] Contralateral; Friel et al., 2021 [24] Ipsilateral; Sakzweski et al., 2011 [23]; Sakzweski et al., 2015 [22]
		COPM Satisfaction	Brandao et al., 2012 [21]; Friel et al., 2021 [24] Bilateral; Friel et al., 2021 [24] Contralateral; Friel et al., 2021 [24] Ipsilateral; Sakzweski et al., 2015 [22]
		PEDI functional skills	Brandao et al., 2012 [21]; Friel et al., 2021 [24] Bilateral; Friel et al., 2021 [24] Contralateral; Friel et al., 2021 [24] Ipsilateral
		PEDI independence	Brandao et al., 2012 [21]; Friel et al., 2021 [24] Bilateral; Friel et al., 2021 [24] Contralateral; Friel et al., 2021 [24] Ipsilateral
Short term	Unimanual ULF	QUEST dissociate domain	Bingöl et al., 2022 [27]; Gelkop et al., 2015 [29]; Gordon et al., 2011 [20]
		QUEST grasp domain	Bingöl et al., 2022 [27]; Fedrizzi et al., 2013 [25]; Gelkop et al., 2015 [29]; Gordon et al., 2011 [20]
		QUEST total score	Fedrizzi et al., 2013 [25]; Gelkop et al., 2015 [29]
		JTHFT	Gordon et al., 2011 [20]; Sakzweski et al., 2011 [23]; Sakzweski et al., 2015 [22]
	Bimanual ULF	AHA	Gelkop et al., 2015 [29]; Sakzweski et al., 2011 [23]; Sakzweski et al., 2015 [22]
Long term	Unimanual ULF	QUEST grasp domain	Fedrizzi et al., 2013 [25]; Gordon et al., 2011 [20]
		JTHFT	Friel et al., 2021 [24] Bilateral; Friel et al., 2021 [24] Contralateral; Friel et al., 2021 [24] Ipsilateral; Gordon et al., 2011 [20]; Sakzweski et al., 2011 [23]; Sakzweski et al., 2015 [22]
	Bimanual ULF	AHA	Friel et al., 2021 [24] Bilateral; Friel et al., 2021 [24] Contralateral; Friel et al., 2021 [24] Ipsilateral; Sakzweski et al., 2011 [23]; Sakzweski et al., 2015 [22]

## Data Availability

The data collected are already in the public domain.

## Supplementary Materials

The following supporting information can be downloaded at: https://www.mdpi.com/article/10.3390/children12060804/s1, Table S1: PRISMA 2020 Checklist; Table S2: Search strategy; Figure S1: Information flow diagram of the selection process for the systematic review and meta-analysis; Table S3: Reasons for exclusion after full-text revision of potential studies; Figures S2: Funnel plots for the risk of bias in the meta-analyses; Figures S3: Sensitivity analyses for the papers with higher risk of bias.

## Abbreviations

The following abbreviations were used in this manuscript:

ADL	Activities of daily living
AHA	Assisting Hand Assessment
BIT	Bimanual training
CIMT	Constraint-induced movement therapy
COPM	Canadian Occupational Performance Measure
CP	Cerebral palsy
HABIT	Hand–Arm Bimanual Intensive Training
JTHFT	Jebsen-Taylor Hand Function Test
mCIMT	Modified constraint-induced movement therapy
PEDI	Paediatric Evaluation of Disability Inventory
QUEST	Quality of Upper Extremity Test
RCT	Randomised controlled trial
ULF	Upper limb function
