# Supplementary material for: Constraint-Induced Movement Therapy Versus Bimanual Training to Improve Upper Limb Function in Cerebral Palsy: A Systematic Review and Meta-Analysis of Follow-Ups
Source: Children (Basel). 2025 Jun 19;12(6):804. doi: 10.3390/children12060804 (PMC12191506; doi:10.3390/children12060804)
Supplement: Supplementary file 1 [file children-12-00804-s001.zip › Supplementary Figure S1. Flow diagram.pdf]

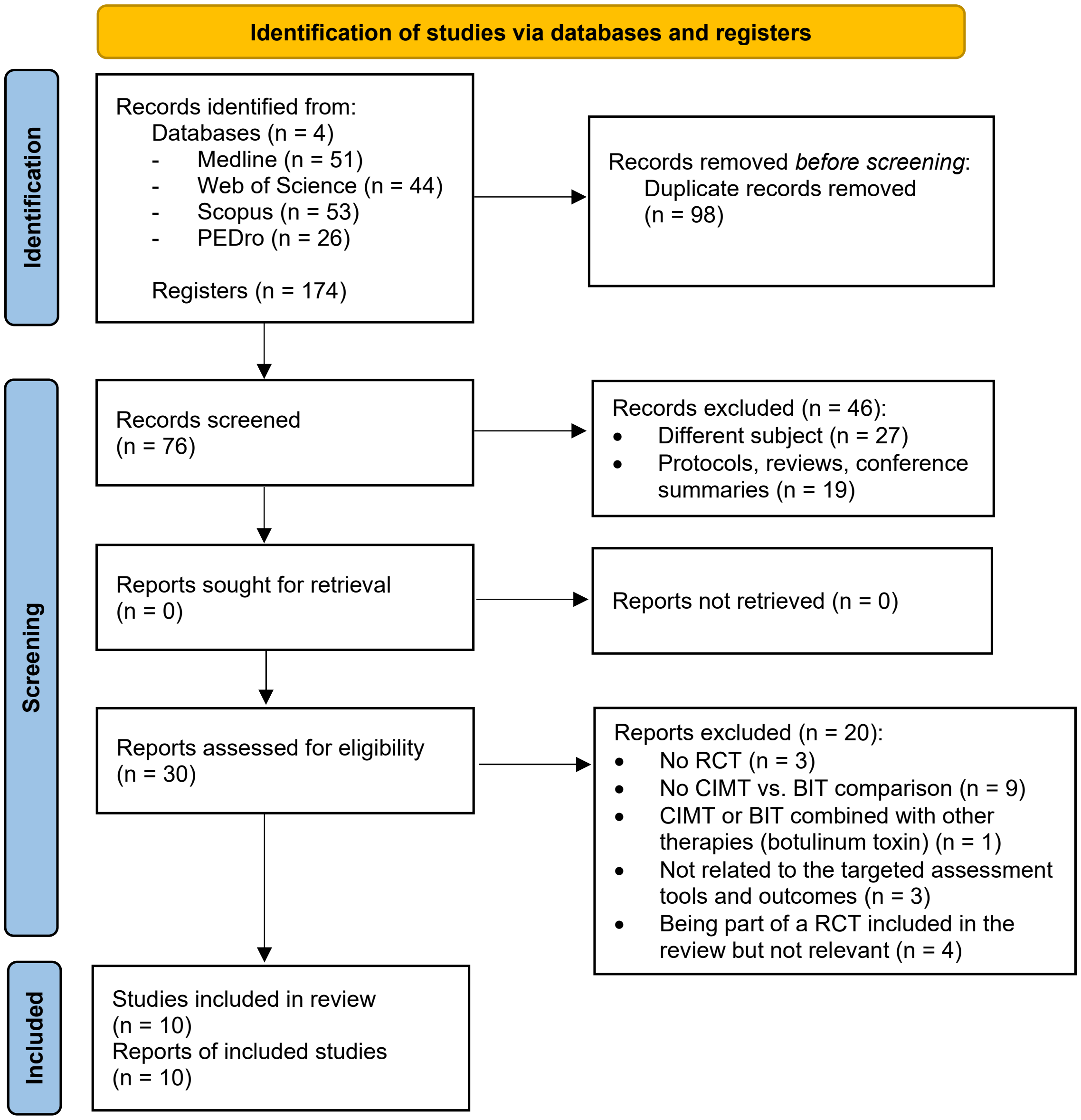

Supplementary Figure S1. Information flow diagram of the selection process for the systematic review and meta-analysis.
