# Supplementary material for: Constraint-Induced Movement Therapy Versus Bimanual Training to Improve Upper Limb Function in Cerebral Palsy: A Systematic Review and Meta-Analysis of Follow-Ups
Source: Children (Basel). 2025 Jun 19;12(6):804. doi: 10.3390/children12060804 (PMC12191506; doi:10.3390/children12060804)
Supplement: Supplementary file 1 [file children-12-00804-s001.zip › Supplementary Figures S2. Publication bias.pdf]

Supplementary Figures S2. Funnel plots for the risk of bias in the meta-analyses.

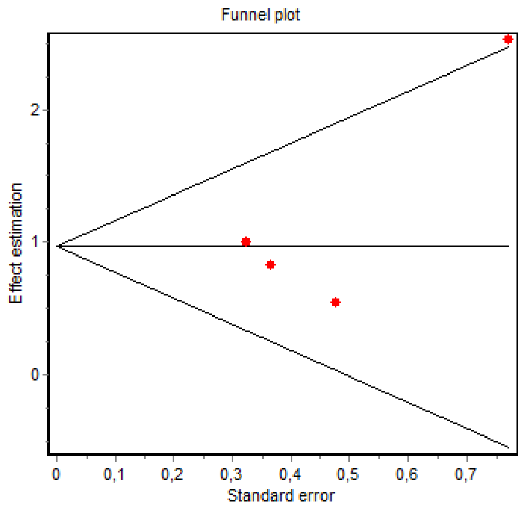

Fig. S.2.1. Funnel plot for Figure 3A. Immediate-term results for unimanual upper limb function measured by QUEST and JTHFT: CIMT vs. BIT on QUEST dissociated movement domain.

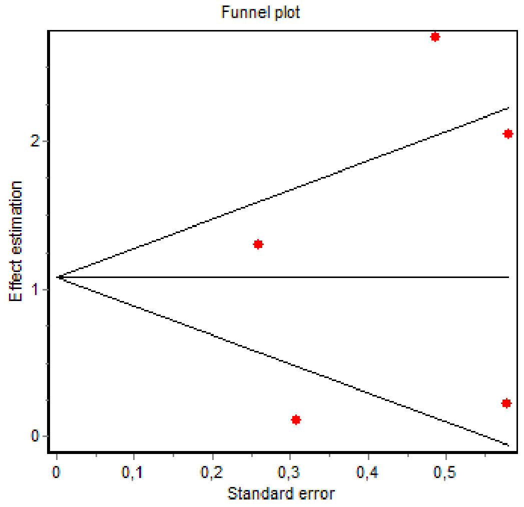

Fig. S.2.2. Funnel plot for Figure 3B. Immediate-term results for unimanual upper limb function measured by QUEST and JTHFT: CIMT vs. BIT on QUEST grasp domain.

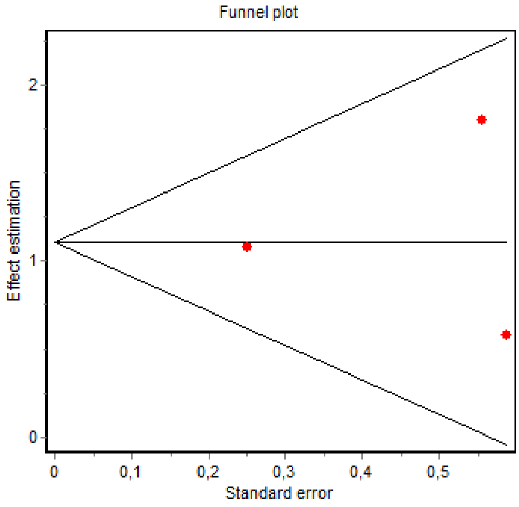

Fig. S.2.3. Funnel plot for Figure 3C. Immediate-term results for unimanual upper limb function measured by QUEST and JTHFT: CIMT vs. BIT on QUEST total.

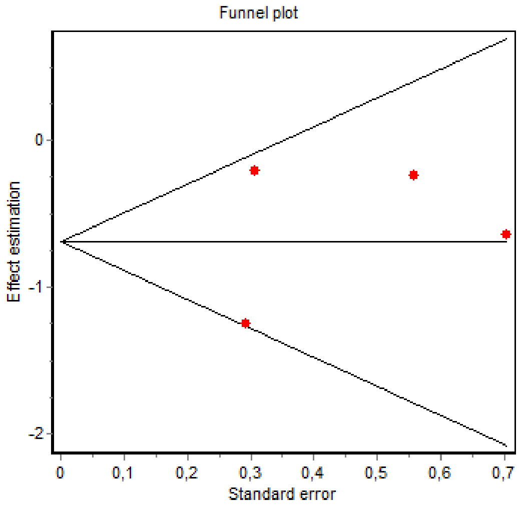

Fig. S.2.4. Funnel plot for Figure 3D. Immediate-term results for unimanual upper limb function measured by QUEST and JTHFT: CIMT vs. BIT on JTHFT.

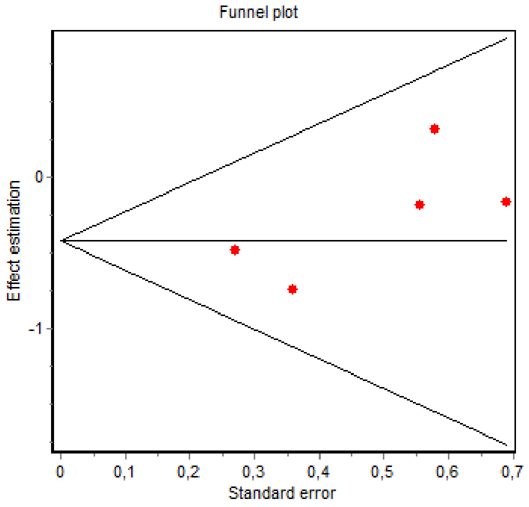

Fig. S.2.5. Funnel plot for Figure 4. Immediate-term results for bimanual upper limb function measured by AHA.

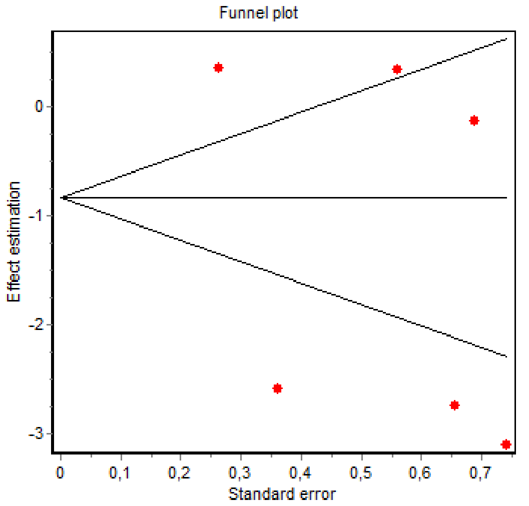

Fig. S.2.6. Funnel plot for Figure 5A. Immediate-term results for occupational performance and disability measured by COPM and PEDI: CIMT vs. BIT on the COPM performance domain.

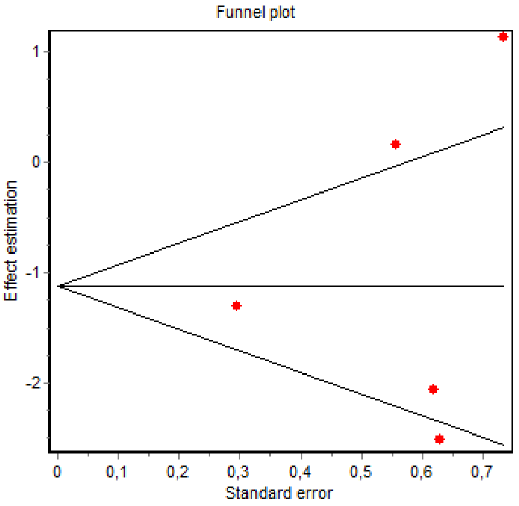

Fig. S.2.7. Funnel plot for Figure 5B. Immediate-term results for occupational performance and disability measured by COPM and PEDI: CIMT vs. BIT on COPM satisfaction domain.

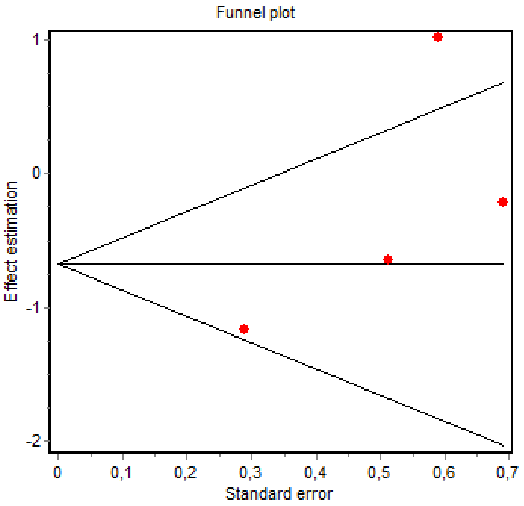

Fig. S.2.8. Funnel plot for Figure 5C. Immediate-term results for occupational performance and disability measured by COPM and PEDI: CIMT vs. BIT on PEDI functional skills domain.

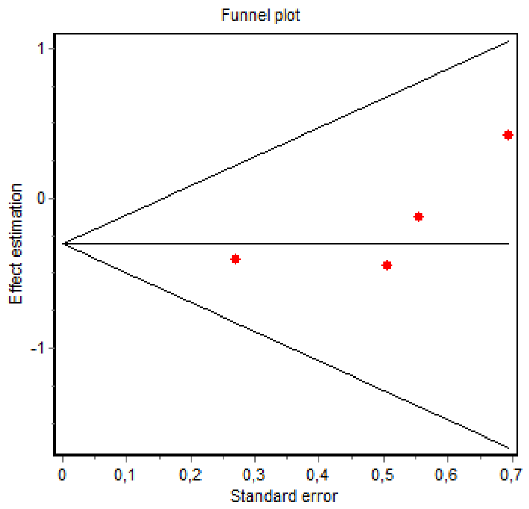

Fig. S.2.9. Funnel plot for Figure 5D. Immediate-term results for occupational performance and disability measured by COPM and PEDI: CIMT vs. BIT on PEDI independence domain.

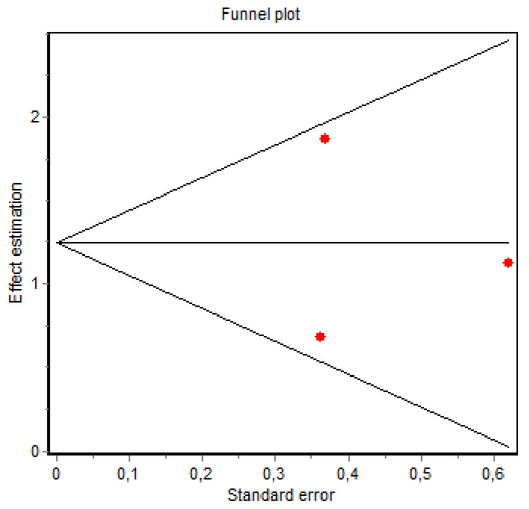

Fig. S.2.10. Funnel plot for Figure 6A. Short-term results for unimanual upper limb function measured by QUEST and JTHFT: CIMT vs. BIT on QUEST dissociated movement domain.

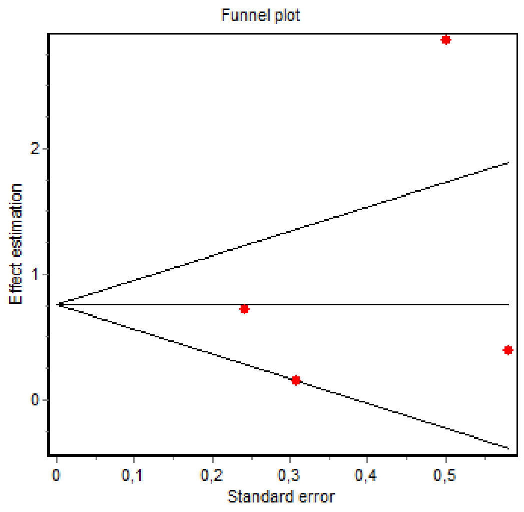

Fig. S.2.11. Funnel plot for Figure 6B. Short-term results for unimanual upper limb function measured by QUEST and JTHFT: CIMT vs. BIT on QUEST grasp domain.

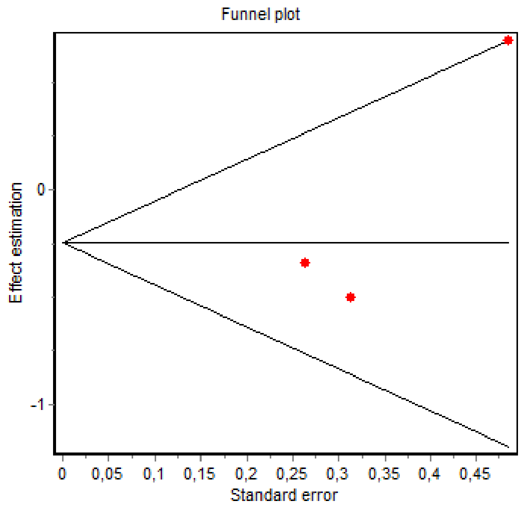

Fig. S.2.12. Funnel plot for Figure 6D. Short-term results for unimanual upper limb function measured by QUEST and JTHFT: CIMT vs. BIT on JTHFT.

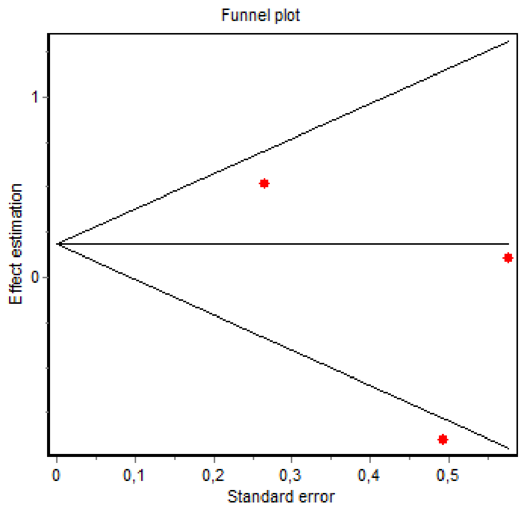

Fig. S.2.13. Funnel plot for Figure 7. Short-term results for bimanual upper limb function measured by AHA.

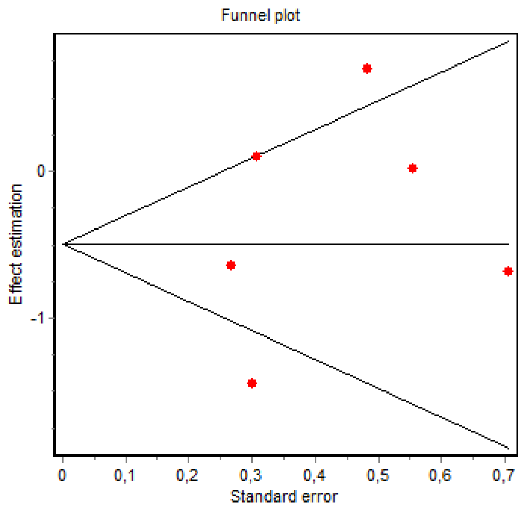

Fig. S.2.14. Funnel plot for Figure 8B. Long-term results for unimanual upper limb function measured by QUEST and JTHFT: CIMT vs. BIT on JTHFT.

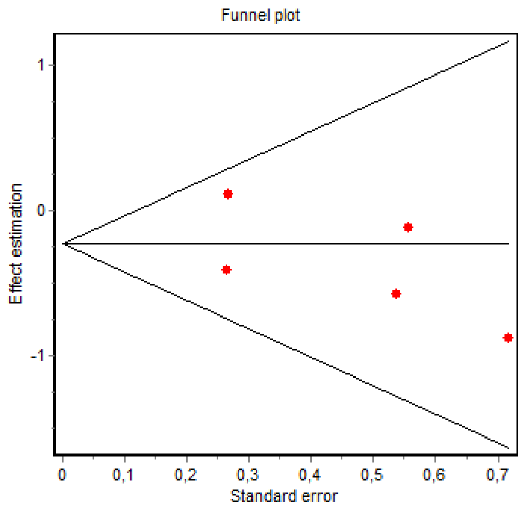

Fig. S.2.15. Funnel plot for Figure 9. Long-term results for bimanual upper limb function measured by AHA.

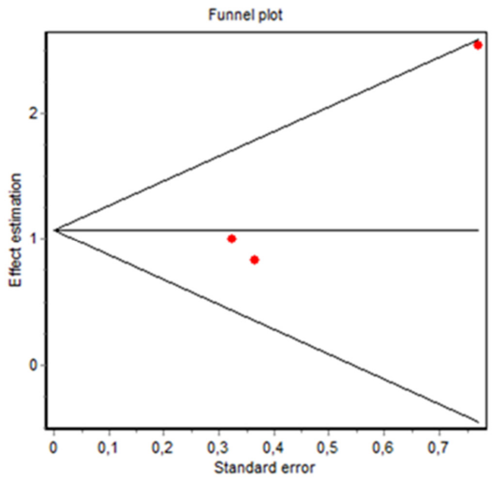

Fig. S.2.16. Funnel plot for Figure 10A. Total intervention hours (90>h) for unimanual limb function measured by QUEST dissociated movement domain.

Formatted: Font: (Default) Times New Roman, (Asian) Times New Roman, English (United Kingdom)

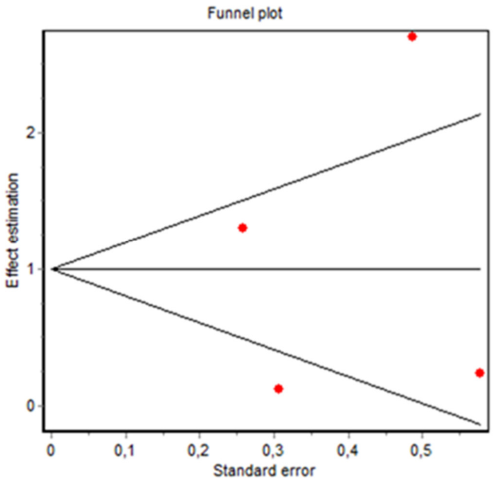

Fig. S.2.17. Funnel plot for Figure 10B. Total intervention hours (90>h) for unimanual limb function measured by QUEST grasp domain.

Formatted: Font: (Default) Times New Roman, (Asian) Times New Roman, English (United Kingdom)
