# Supplementary material for: Constraint-Induced Movement Therapy Versus Bimanual Training to Improve Upper Limb Function in Cerebral Palsy: A Systematic Review and Meta-Analysis of Follow-Ups
Source: Children (Basel). 2025 Jun 19;12(6):804. doi: 10.3390/children12060804 (PMC12191506; doi:10.3390/children12060804)

**Supplementary Figures S3.** Sensitivity analyses for the papers with higher risk of bias.

**Fig. S3.1.** Sensitivity analysis for Figure 3A. Immediate-term results for unimanual upper limb function measured by QUEST and JTHFT: CIMT vs. BIT on QUEST dissociated movement domain.

Original:

| | CIMT | | | BIT | | | | Std. Mean Difference |
|---|---|---|---|---|---|---|---|---|
| Study or Subgroup | Mean | SD | Total | Mean | SD | Total | Weight | IV, Fixed, 95% CI |
| Bingöl et al., 2022 | 13.57 | 2.2766 | 16 | 10.82 | 4.1284 | 16 | 32.7% | 0.80 [0.08, 1.53] |
| Gelkop et al., 2015 | 21 | 6.5812 | 6 | 4.4 | 6.5834 | 6 | 6.7% | 2.33 [0.72, 3.93] |
| Gordon et al., 2011 | 5.1 | 1.611 | 21 | 3.5 | 1.6037 | 21 | 41.4% | 0.98 [0.33, 1.62] |
| Zafer et al., 2016 | 33.5 | 2.3711 | 9 | 32.28 | 2.1657 | 9 | 19.3% | 0.51 [-0.43, 1.45] |
| **Total (95% CI)** | | | **52** | | | **52** | **100.0%** | **0.92 [0.51, 1.33]** |

Heterogeneity: Chi² = 3.81, df = 3 (P = 0.28); I² = 21%
Test for overall effect: Z = 4.36 (P < 0.0001)

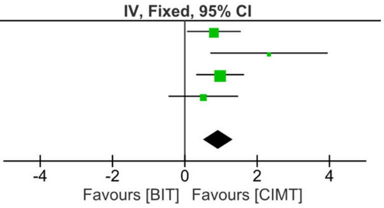


Excluding Zafer et al. 2016:

| | CIMT | | | BIT | | | | Std. Mean Difference |
|---|---|---|---|---|---|---|---|---|
| Study or Subgroup | Mean | SD | Total | Mean | SD | Total | Weight | IV, Fixed, 95% CI |
| Bingöl et al., 2022 | 13.57 | 2.2766 | 16 | 10.82 | 4.1284 | 16 | 40.5% | 0.80 [0.08, 1.53] |
| Gelkop et al., 2015 | 21 | 6.5812 | 6 | 4.4 | 6.5834 | 6 | 8.3% | 2.33 [0.72, 3.93] |
| Gordon et al., 2011 | 5.1 | 1.611 | 21 | 3.5 | 1.6037 | 21 | 51.3% | 0.98 [0.33, 1.62] |
| Zafer et al., 2016 | 33.5 | 2.3711 | 9 | 32.28 | 2.1657 | 9 | 0.0% | 0.51 [-0.43, 1.45] |
| **Total (95% CI)** | | | **43** | | | **43** | **100.0%** | **1.02 [0.56, 1.48]** |

Heterogeneity: Chi² = 2.91, df = 2 (P = 0.23); I² = 31%
Test for overall effect: Z = 4.33 (P < 0.0001)

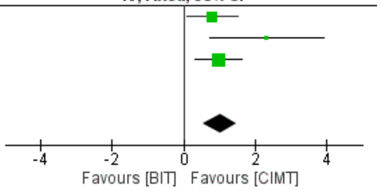


**Fig. S3.2.** Sensitivity analysis for Figure 3B. Immediate-term results for unimanual upper limb function measured by QUEST and JTHFT: CIMT vs. BIT on QUEST grasp domain.

Original:

| | CIMT | | | BIT | | | | Std. Mean Difference |
|---|---|---|---|---|---|---|---|---|
| Study or Subgroup | Mean | SD | Total | Mean | SD | Total | Weight | IV, Random, 95% CI |
| Bingöl et al., 2022 | 15.04 | 3.3503 | 16 | 6.15 | 3.2503 | 16 | 19.2% | 2.63 [1.65, 3.60] |
| Fedrizzi et al. 2013 | 7 | 2.8637 | 39 | 2.7 | 3.7794 | 33 | 23.1% | 1.28 [0.77, 1.79] |
| Gelkop et al., 2015 | 14.1 | 7.978 | 6 | 12.3 | 8.0997 | 6 | 17.8% | 0.21 [-0.93, 1.34] |
| Gordon et al., 2011 | 11.1 | 2.7725 | 21 | 10.8 | 2.7725 | 21 | 22.4% | 0.11 [-0.50, 0.71] |
| Zafer et al., 2016 | 34.77 | 2.0844 | 9 | 30.9 | 1.697 | 9 | 17.5% | 1.94 [0.77, 3.11] |
| **Total (95% CI)** | | | **91** | | | **85** | **100.0%** | **1.20 [0.31, 2.09]** |

Heterogeneity: Tau² = 0.83; Chi² = 24.49, df = 4 (P < 0.0001); I² = 84%
Test for overall effect: Z = 2.64 (P = 0.008)

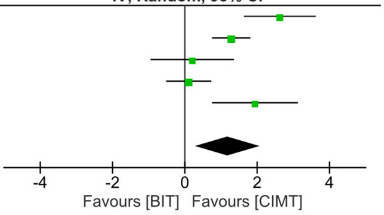


Excluding Zafer et al. 2016:

| | CIMT | | | BIT | | | | Std. Mean Difference |
|---|---|---|---|---|---|---|---|---|
| Study or Subgroup | Mean | SD | Total | Mean | SD | Total | Weight | IV, Random, 95% CI |
| Bingöl et al., 2022 | 15.04 | 3.3503 | 16 | 6.15 | 3.2503 | 16 | 23.4% | 2.63 [1.65, 3.60] |
| Fedrizzi et al. 2013 | 7 | 2.8637 | 39 | 2.7 | 3.7794 | 33 | 27.8% | 1.28 [0.77, 1.79] |
| Gelkop et al., 2015 | 14.1 | 7.978 | 6 | 12.3 | 8.0997 | 6 | 21.7% | 0.21 [-0.93, 1.34] |
| Gordon et al., 2011 | 11.1 | 2.7725 | 21 | 10.8 | 2.7725 | 21 | 27.0% | 0.11 [-0.50, 0.71] |
| Zafer et al., 2016 | 34.77 | 2.0844 | 9 | 30.9 | 1.697 | 9 | 0.0% | 1.94 [0.77, 3.11] |
| **Total (95% CI)** | | | **82** | | | **76** | **100.0%** | **1.05 [0.03, 2.06]** |

Heterogeneity: Tau² = 0.90; Chi² = 22.06, df = 3 (P < 0.0001); I² = 86%
Test for overall effect: Z = 2.02 (P = 0.04)

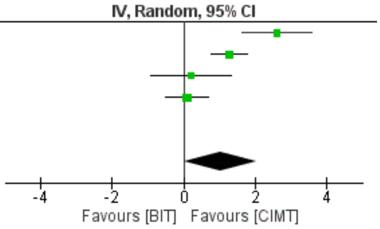

**Fig. S3.3.** Sensitivity analysis for Figure 3C. Immediate-term results for unimanual upper limb function measured by QUEST and JTHFT: CIMT vs. BIT on QUEST total.

Original:

| | CIMT | | | BIT | | | | Std. Mean Difference |
|---|---|---|---|---|---|---|---|---|
| Study or Subgroup | Mean | SD | Total | Mean | SD | Total | Weight | IV, Fixed, 95% CI |
| Fedrizzi et al. 2013 | 6.9 | 2.4611 | 39 | 3.7 | 3.5338 | 33 | 72.5% | 1.06 [0.56, 1.55] |
| Gelkop et al., 2015 | 19 | 9.4475 | 6 | 13.6 | 9.4475 | 6 | 13.3% | 0.53 [-0.63, 1.69] |
| Zafer et al., 2016 | 21.07 | 1.541 | 9 | 18.7 | 1.0702 | 9 | 14.3% | 1.70 [0.58, 2.82] |
| **Total (95% CI)** | | | **54** | | | **48** | **100.0%** | **1.08 [0.66, 1.50]** |

Heterogeneity: Chi² = 2.07, df = 2 (P = 0.36); I² = 3%
Test for overall effect: Z = 5.00 (P < 0.00001)

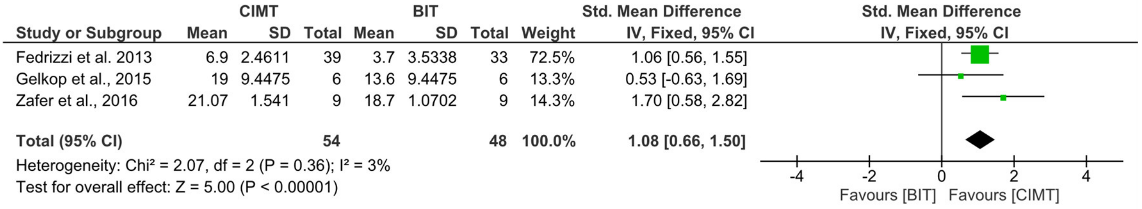


Excluding Zafer et al. 2016:

| | CIMT | | | BIT | | | | Std. Mean Difference |
|---|---|---|---|---|---|---|---|---|
| Study or Subgroup | Mean | SD | Total | Mean | SD | Total | Weight | IV, Fixed, 95% CI |
| Fedrizzi et al. 2013 | 6.9 | 2.4611 | 39 | 3.7 | 3.5338 | 33 | 84.5% | 1.06 [0.56, 1.55] |
| Gelkop et al., 2015 | 19 | 9.4475 | 6 | 13.6 | 9.4475 | 6 | 15.5% | 0.53 [-0.63, 1.69] |
| Zafer et al., 2016 | 21.07 | 1.541 | 9 | 18.7 | 1.0702 | 9 | 0.0% | 1.70 [0.58, 2.82] |
| **Total (95% CI)** | | | **45** | | | **39** | **100.0%** | **0.97 [0.52, 1.43]** |

Heterogeneity: Chi² = 0.67, df = 1 (P = 0.41); I² = 0%
Test for overall effect: Z = 4.18 (P < 0.0001)

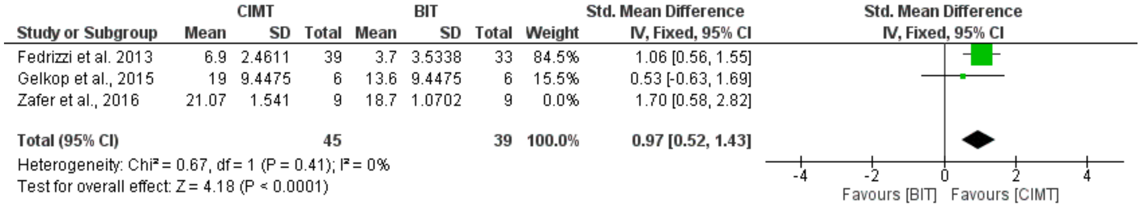


**Fig. S3.4.** Sensitivity analysis for Figure 5A. Immediate-term results for occupational performance and disability measured by COPM and PEDI: CIMT vs. BIT on the COPM performance domain.

Original:

| | CIMT | | | BIT | | | | Std. Mean Difference |
|---|---|---|---|---|---|---|---|---|
| Study or Subgroup | Mean | SD | Total | Mean | SD | Total | Weight | IV, Random, 95% CI |
| Brandao et al., 2012 | 1.59 | 0.5394 | 8 | 3.02 | 0.3651 | 8 | 15.2% | -2.94 [-4.46, -1.41] |
| Friel et al., 2021 Bilateral | 3.1 | 0.492 | 7 | 2.9 | 0.7143 | 6 | 16.8% | 0.31 [-0.79, 1.41] |
| Friel et al., 2021 Contralateral | 2.3 | 0.7516 | 7 | 2.4 | 0.5681 | 3 | 15.9% | -0.13 [-1.48, 1.23] |
| Friel et al., 2021 Ipsilateral | 2.8 | 0.3103 | 26 | 3.6 | 0.3061 | 30 | 17.9% | -2.56 [-3.28, -1.84] |
| Sakzewski et al., 2011 | 2.9 | 0.3057 | 29 | 2.8 | 0.2553 | 29 | 18.3% | 0.35 [-0.17, 0.87] |
| Sakzewski et al., 2015 | 1.8 | 0.4682 | 9 | 3.1 | 0.4799 | 9 | 15.9% | -2.61 [-3.95, -1.28] |
| **Total (95% CI)** | | | **86** | | | **85** | **100.0%** | **-1.23 [-2.60, 0.14]** |

Heterogeneity: Tau² = 2.60; Chi² = 61.28, df = 5 (P < 0.00001); I² = 92%
Test for overall effect: Z = 1.75 (P = 0.08)

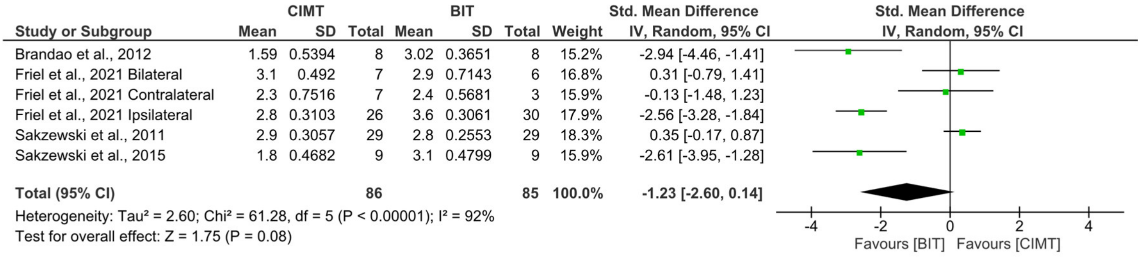


Excluding Brandao et al. 2012:

| | CIMT | | | BIT | | | | Std. Mean Difference |
|---|---|---|---|---|---|---|---|---|
| Study or Subgroup | Mean | SD | Total | Mean | SD | Total | Weight | IV, Random, 95% CI |
| Brandao et al., 2012 | 1.59 | 0.5394 | 8 | 3.02 | 0.3651 | 8 | 0.0% | -2.94 [-4.46, -1.41] |
| Friel et al., 2021 Bilateral | 3.1 | 0.492 | 7 | 2.9 | 0.7143 | 6 | 19.8% | 0.31 [-0.79, 1.41] |
| Friel et al., 2021 Contralateral | 2.3 | 0.7516 | 7 | 2.4 | 0.5681 | 3 | 18.7% | -0.13 [-1.48, 1.23] |
| Friel et al., 2021 Ipsilateral | 2.8 | 0.3103 | 26 | 3.6 | 0.3061 | 30 | 21.1% | -2.56 [-3.28, -1.84] |
| Sakzewski et al., 2011 | 2.9 | 0.3057 | 29 | 2.8 | 0.2553 | 29 | 21.6% | 0.35 [-0.17, 0.87] |
| Sakzewski et al., 2015 | 1.8 | 0.4682 | 9 | 3.1 | 0.4799 | 9 | 18.8% | -2.61 [-3.95, -1.28] |
| **Total (95% CI)** | | | **78** | | | **77** | **100.0%** | **-0.92 [-2.38, 0.55]** |

Heterogeneity: Tau² = 2.51; Chi² = 53.13, df = 4 (P < 0.00001); I² = 92%
Test for overall effect: Z = 1.23 (P = 0.22)

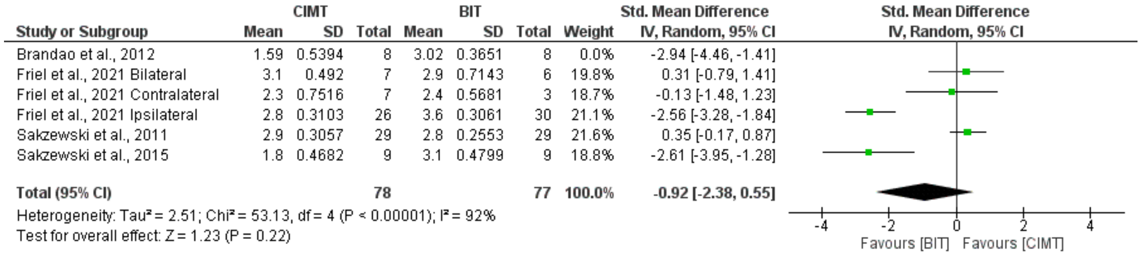

**Fig. S3.5.** Sensitivity analysis for Figure 5B. Immediate-term results for occupational performance and disability measured by COPM and PEDI: CIMT vs. BIT on COPM satisfaction domain.

Original:

| Study or Subgroup | CIMT Mean | CIMT SD | CIMT Total | BIT Mean | BIT SD | BIT Total | Weight | Std. Mean Difference IV, Random, 95% CI |
|---|---|---|---|---|---|---|---|---|
| Brandao et al., 2012 | 1.54 | 0.656 | 8 | 2.75 | 0.5034 | 8 | 19.1% | -1.96 [-3.21, -0.70] |
| Friel et al., 2021 Bilateral | 4.2 | 0.6393 | 7 | 4.1 | 0.575 | 6 | 20.4% | 0.15 [-0.94, 1.24] |
| Friel et al., 2021 Contralateral | 2.8 | 0.8852 | 7 | 1.7 | 1.1998 | 3 | 17.3% | 1.02 [-0.45, 2.49] |
| Friel et al., 2021 Ipsilateral | 3.2 | 0.405 | 26 | 3.7 | 0.3571 | 30 | 24.3% | -1.30 [-1.88, -0.72] |
| Sakzewski et al., 2015 | 1.9 | 0.4649 | 9 | 3.5 | 0.7716 | 9 | 18.9% | -2.39 [-3.67, -1.11] |
| **Total (95% CI)** | | | **57** | | | **56** | **100.0%** | **-0.93 [-1.98, 0.12]** |

Heterogeneity: Tau² = 1.10; Chi² = 19.20, df = 4 (P = 0.0007); I² = 79%
Test for overall effect: Z = 1.74 (P = 0.08)

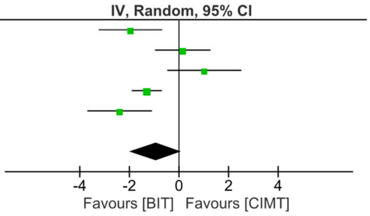


Excluding Brandao et al. 2012:

| Study or Subgroup | CIMT Mean | CIMT SD | CIMT Total | BIT Mean | BIT SD | BIT Total | Weight | Std. Mean Difference IV, Random, 95% CI |
|---|---|---|---|---|---|---|---|---|
| Brandao et al., 2012 | 1.54 | 0.656 | 8 | 2.75 | 0.5034 | 8 | 0.0% | -1.96 [-3.21, -0.70] |
| Friel et al., 2021 Bilateral | 4.2 | 0.6393 | 7 | 4.1 | 0.575 | 6 | 25.3% | 0.15 [-0.94, 1.24] |
| Friel et al., 2021 Contralateral | 2.8 | 0.8852 | 7 | 1.7 | 1.1998 | 3 | 21.9% | 1.02 [-0.45, 2.49] |
| Friel et al., 2021 Ipsilateral | 3.2 | 0.405 | 26 | 3.7 | 0.3571 | 30 | 29.3% | -1.30 [-1.88, -0.72] |
| Sakzewski et al., 2015 | 1.9 | 0.4649 | 9 | 3.5 | 0.7716 | 9 | 23.6% | -2.39 [-3.67, -1.11] |
| **Total (95% CI)** | | | **49** | | | **48** | **100.0%** | **-0.68 [-1.93, 0.57]** |

Heterogeneity: Tau² = 1.30; Chi² = 17.07, df = 3 (P = 0.0007); I² = 82%
Test for overall effect: Z = 1.07 (P = 0.29)

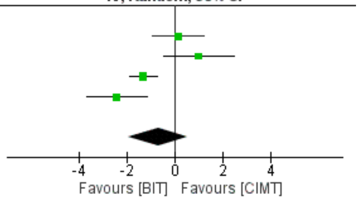


**Fig. S3.6.** Sensitivity analysis for Figure 5C. Immediate-term results for occupational performance and disability measured by COPM and PEDI: CIMT vs. BIT on PEDI functional skills domain.

Original:

| Study or Subgroup | CIMT Mean | CIMT SD | CIMT Total | BIT Mean | BIT SD | BIT Total | Weight | Std. Mean Difference IV, Random, 95% CI |
|---|---|---|---|---|---|---|---|---|
| Brandao et al., 2012 | 2 | 2.2375 | 8 | 3.38 | 1.9967 | 8 | 25.2% | -0.62 [-1.63, 0.39] |
| Friel et al., 2021 Bilateral | 4.2 | 2.6589 | 7 | 1.5 | 2.6997 | 6 | 22.8% | 0.94 [-0.23, 2.11] |
| Friel et al., 2021 Contralateral | 1 | 1.4313 | 7 | 1.4 | 2.5749 | 3 | 20.2% | -0.20 [-1.56, 1.16] |
| Friel et al., 2021 Ipsilateral | 3.1 | 1.0356 | 26 | 4.4 | 1.169 | 30 | 31.8% | -1.16 [-1.73, -0.59] |
| **Total (95% CI)** | | | **48** | | | **47** | **100.0%** | **-0.35 [-1.27, 0.57]** |

Heterogeneity: Tau² = 0.61; Chi² = 10.48, df = 3 (P = 0.01); I² = 71%
Test for overall effect: Z = 0.75 (P = 0.46)

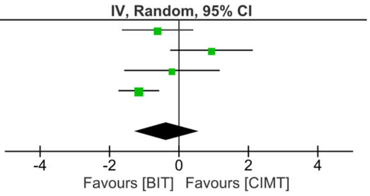


Excluding Brandao et al. 2012:

| Study or Subgroup | CIMT Mean | CIMT SD | CIMT Total | BIT Mean | BIT SD | BIT Total | Weight | Std. Mean Difference IV, Random, 95% CI |
|---|---|---|---|---|---|---|---|---|
| Brandao et al., 2012 | 2 | 2.2375 | 8 | 3.38 | 1.9967 | 8 | 0.0% | -0.62 [-1.63, 0.39] |
| Friel et al., 2021 Bilateral | 4.2 | 2.6589 | 7 | 1.5 | 2.6997 | 6 | 31.7% | 0.94 [-0.23, 2.11] |
| Friel et al., 2021 Contralateral | 1 | 1.4313 | 7 | 1.4 | 2.5749 | 3 | 29.3% | -0.20 [-1.56, 1.16] |
| Friel et al., 2021 Ipsilateral | 3.1 | 1.0356 | 26 | 4.4 | 1.169 | 30 | 39.0% | -1.16 [-1.73, -0.59] |
| **Total (95% CI)** | | | **40** | | | **39** | **100.0%** | **-0.21 [-1.55, 1.13]** |

Heterogeneity: Tau² = 1.11; Chi² = 10.46, df = 2 (P = 0.005); I² = 81%
Test for overall effect: Z = 0.31 (P = 0.76)

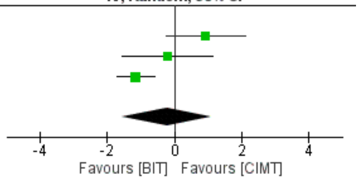

**Fig. S3.7.** Sensitivity analysis for Figure 5D. Immediate-term results for occupational performance and disability measured by COPM and PEDI: CIMT vs. BIT on PEDI independence domain.

Original:

| Study or Subgroup | CIMT Mean | CIMT SD | CIMT Total | BIT Mean | BIT SD | BIT Total | Weight | Std. Mean Difference IV, Fixed, 95% CI |
|---|---|---|---|---|---|---|---|---|
| Brandao et al., 2012 | 0.25 | 2.6047 | 8 | 1.25 | 1.645 | 8 | 17.0% | -0.43 [-1.43, 0.56] |
| Friel et al., 2021 Bilateral | 0.9 | 1.9179 | 7 | 1.2 | 2.5875 | 6 | 14.1% | -0.12 [-1.22, 0.97] |
| Friel et al., 2021 Contralateral | 1.2 | 1.5109 | 7 | 0 | 5.2103 | 3 | 9.0% | 0.37 [-1.00, 1.74] |
| Friel et al., 2021 Ipsilateral | 1.9 | 1.069 | 26 | 2.3 | 0.8522 | 30 | 59.8% | -0.41 [-0.94, 0.12] |
| **Total (95% CI)** | | | **48** | | | **47** | **100.0%** | **-0.30 [-0.71, 0.11]** |

Heterogeneity: Chi² = 1.26, df = 3 (P = 0.74); I² = 0%
Test for overall effect: Z = 1.45 (P = 0.15)

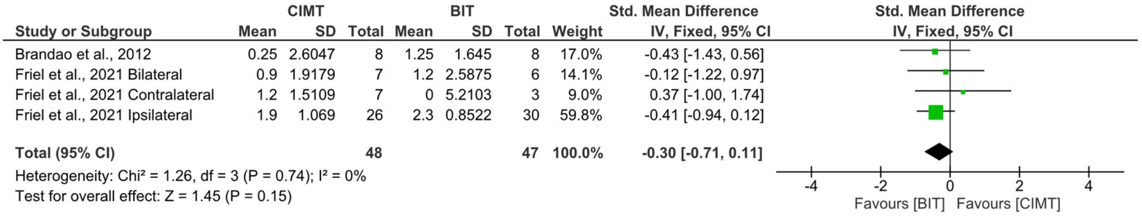


Excluding Brandao et al. 2012:

| Study or Subgroup | CIMT Mean | CIMT SD | CIMT Total | BIT Mean | BIT SD | BIT Total | Weight | Std. Mean Difference IV, Fixed, 95% CI |
|---|---|---|---|---|---|---|---|---|
| Brandao et al., 2012 | 0.25 | 2.6047 | 8 | 1.25 | 1.645 | 8 | 0.0% | -0.43 [-1.43, 0.56] |
| Friel et al., 2021 Bilateral | 0.9 | 1.9179 | 7 | 1.2 | 2.5875 | 6 | 17.1% | -0.12 [-1.22, 0.97] |
| Friel et al., 2021 Contralateral | 1.2 | 1.5109 | 7 | 0 | 5.2103 | 3 | 10.9% | 0.37 [-1.00, 1.74] |
| Friel et al., 2021 Ipsilateral | 1.9 | 1.069 | 26 | 2.3 | 0.8522 | 30 | 72.1% | -0.41 [-0.94, 0.12] |
| **Total (95% CI)** | | | **40** | | | **39** | **100.0%** | **-0.28 [-0.73, 0.17]** |

Heterogeneity: Chi² = 1.18, df = 2 (P = 0.55); I² = 0%
Test for overall effect: Z = 1.21 (P = 0.23)

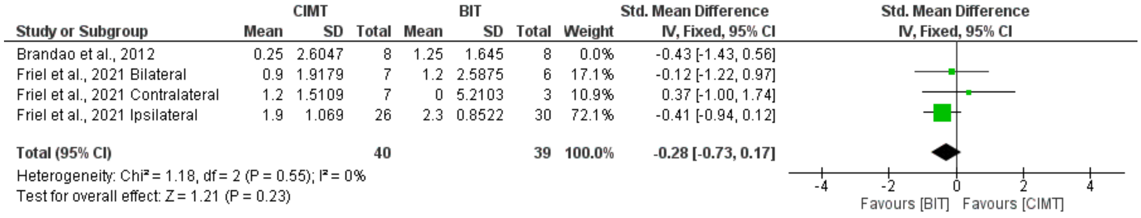

Supplement: Supplementary file 1 [file children-12-00804-s001.zip › Supplementary Figures S3. Sensitivity analyses.pdf]
