# Supplementary material for: Constraint-Induced Movement Therapy Versus Bimanual Training to Improve Upper Limb Function in Cerebral Palsy: A Systematic Review and Meta-Analysis of Follow-Ups
Source: Children (Basel). 2025 Jun 19;12(6):804. doi: 10.3390/children12060804 (PMC12191506; doi:10.3390/children12060804)
Supplement: Supplementary file 1 [file children-12-00804-s001.zip › Supplementary Table S2. Search Strategy.pdf]

Supplementary Table S2. Search strategy.

Database	PubMed
Search terms	"Cerebral Palsy"[Mesh] AND ((constrain* AND (movement* OR therap*)) OR CIMT OR mCIMT OR "CI therap*" OR unilateral OR unimanual) AND (bimanual OR BIT OR BIM OR HABIT)
Filters	Randomized Controlled Trial
Results	51 records

Database	Scopus
Search terms	"cerebral palsy" AND (constrain* OR CIMT OR mCIMT OR CI therap* OR unilateral OR unimanual) AND (bimanual OR BIT OR BIM OR HABIT) AND ("randomized clinical trial" OR "randomized controlled trial" OR "clinical trial")
Filters	Articles
Results	53 records

Database	Web of Science
Search terms	"cerebral palsy" AND (constrain* OR CIMT OR mCIMT OR CI therap* OR unilateral OR unimanual) AND (bimanual OR BIT OR BIM OR HABIT) AND ("randomized clinical trial" OR "randomized controlled trial" OR "clinical trial")
Filters	Articles
Results	44 records

Database	PEDro
Search terms	A combination of the following terms was made in different searches: "cerebral palsy", “constrain*”, “CIMT”, “mCIMT”, “CI therapy”, “unilateral”, “unimanual”, “bimanual”, “BIT”, “BIM”, “HABIT”.
Filters	Cerebral palsy (Topic); Clinical trial (Method)
Results	26 records
