# Supplementary material for: Constraint-Induced Movement Therapy Versus Bimanual Training to Improve Upper Limb Function in Cerebral Palsy: A Systematic Review and Meta-Analysis of Follow-Ups
Source: Children (Basel). 2025 Jun 19;12(6):804. doi: 10.3390/children12060804 (PMC12191506; doi:10.3390/children12060804)
Supplement: Supplementary file 1 [file children-12-00804-s001.zip › Supplementary Table S3. Reasons for exclusion.pdf]

Supplementary Table S3. Reasons for exclusion after full-text revision of potential studies.

**Study designs not corresponding to randomized controlled trials** (n=3)	Cohen-Holzer M, Katz-Leurer M, Meyer S, Green D, Parush S. The Effect of Bimanual Training with or Without Constraint on Hand Functions in Children with Unilateral Cerebral Palsy: A Non-Randomized Clinical Trial. *Phys Occup Ther Pediatr* 2017; **37**: 516–27.
	Gordon AM, Chinnan A, Gill S, Petra E, Hung YC, Charles J. Both constraint-induced movement therapy and bimanual training lead to improved performance of upper extremity function in children with hemiplegia. *Dev Med Child Neurol* 2008; **50**: 957–8.
	Robert MT, Gutterman J, Ferre CL, Chin K, Brandao MB, Gordon AM, et al. Corpus Callosum Integrity Relates to Improvement of Upper-Extremity Function Following Intensive Rehabilitation in Children With Unilateral Spastic Cerebral Palsy. *Neurorehabil Neural Repair* 2021; **35**: 534–44.
**Combination of CIMT and BIT in at least one of the intervention groups** (n=9)	Deppe W, Thuemmler K, Fleischer J, Berger C, Meyer S, Wiedemann B. Modified constraint-induced movement therapy versus intensive bimanual training for children with hemiplegia-a randomized controlled trial. *Clin Rehabil* 2013; **27**: 909–20.
	Aarts PB, Jongerius PH, Geerdink YA, Van Limbeek J, Geurts AC. Effectiveness of modified constraint-induced movement therapy in children with unilateral spastic cerebral palsy: A randomized controlled trial. *Neurorehabil Neural Repair* 2010; **24**: 509–18.
	Geerdink Y, Aarts P, Geurts AC. Motor learning curve and long-term effectiveness of modified constraint-induced movement therapy in children with unilateral cerebral palsy: A randomized controlled trial. *Res Dev Disabil* 2013; **34**: 923–31.
	De Brito Brandão M, Mancini MC, Vaz DV, Pereira De Melo AP, Fonseca ST. Adapted version of constraint-induced movement therapy promotes functioning in children with cerebral palsy: A randomized controlled trial. *Clin Rehabil* 2010; **24**: 639–47.
	Palomo-Carrión R, Lirio-Romero C, Ferri-Morales A, Jovellar-Isiegas P, Cortés-Vega MD, Romay-Barrero H. Combined intensive therapies at home in spastic unilateral cerebral palsy with high bimanual functional performance. What do they offer? A comparative randomised clinical trial. *Ther Adv Chronic Dis.* 2021; **12**: 1–19.
	Sakzewski L, Miller L, Ziviani J, Abbott DF, Rose S, Macdonell RAL, et al. Randomized comparison trial of density and context of upper limb intensive group versus individualized occupational therapy for children with unilateral cerebral palsy. *Dev Med Child Neurol* 2015; **57**: 539–47.

	Brauers L, Geijen MME, Speth LAWM, Rameckers EAA. Does intensive upper limb treatment modality Hybrid Constrained Induced Movement Therapy (H-CIMT) improve grip and pinch strength or fatigability of the affected hand? *J Pediatr Rehabil Med* 2017; **10**: 11–7.
	Klingels K, Feys H, Molenaers G, et al. Randomized trial of modified constraint-induced movement therapy with and without an intensive therapy program in children with unilateral cerebral palsy. *Neurorehabil Neural Repair* 2013; **27**: 799–807.
	Maitre NL, Jeanvoine A, Yoder PJ, et al. Kinematic and Somatosensory Gains in Infants with Cerebral Palsy After a Multi-Component Upper-Extremity Intervention: A Randomized Controlled Trial. *Brain Topogr* 2020; **33**: 751-766.
**Combination of CIMT and BIT with other therapies (botulinum toxin)** (n=1)	Hoare B, Imms C, Villanueva E, Rawicki HB, Matyas T, Carey L. Intensive therapy following upper limb botulinum toxin A injection in young children with unilateral cerebral palsy: A randomized trial. *Dev Med Child Neurol* 2013; **55**: 238–47.
**The assessment tool differed from the targeted for this review** (n=3)	Hung YC, Spingarn A, Friel KM, Gordon AM. Intensive Unimanual Training Leads to Better Reaching and Head Control than Bimanual Training in Children with Unilateral Cerebral Palsy. *Phys Occup Ther Pediatr* 2020; **40**: 491–505.
	Hung YC, Shirzad F, Saleem M, Gordon AM. Intensive upper extremity training improved whole body movement control for children with unilateral spastic cerebral palsy. *Gait Posture* 2020; **81**: 67–72.
	Hung YC, Casertano L, Hillman A, Gordon AM. The effect of intensive bimanual training on coordination of the hands in children with congenital hemiplegia. *Res Dev Disabil* 2011; **32**: 2724–31.
**Studies referred to data of others which have been already included for being more relevant or studies from two different studies by the same author** (n=4)	Facchin P, Rosa-Rizzotto M, Dalla Pozza LV, et al. Multisite trial comparing the efficacy of constraint-induced movement therapy with that of bimanual intensive training in children with hemiplegic cerebral palsy: Postintervention results. *Am J Phys Med Rehabil* 2011; **90**: 539–53.
	Sakzewski L, Ziviani J, Boyd RN. Best Responders After Intensive Upper-Limb Training for Children With Unilateral Cerebral Palsy. *Arch Phys Med Rehabil* 2011; **92**: 578–84.
	Sakzewski L, Ziviani J, Abbott DF, Macdonell RAL, Jackson GD, Boyd RN. Randomized trial of constraint-induced movement therapy and bimanual training on activity outcomes for children with congenital hemiplegia. *Dev Med Child Neurol* 2011; **53**: 313–20.

	Sakzewski L, Carlon S, Shields N, Ziviani J, Ware RS, Boyd RN. Impact of intensive upper limb rehabilitation on quality of life: A randomized trial in children with unilateral cerebral palsy. *Dev Med Child Neurol* 2012; **54**: 415–23.

Sakzewski L, Carlon S, Shields N, Ziviani J, Ware RS, Boyd RN. Impact of intensive upper limb rehabilitation on quality of life: A randomized trial in children with unilateral cerebral palsy. *Dev Med Child Neurol* 2012; **54**: 415–23.
